# Behavioral and brain responses to verbal stimuli reveal transient periods of cognitive integration of the external world during sleep

**DOI:** 10.1038/s41593-023-01449-7

**Published:** 2023-10-12

**Authors:** Başak Türker, Esteban Munoz Musat, Emma Chabani, Alexandrine Fonteix-Galet, Jean-Baptiste Maranci, Nicolas Wattiez, Pierre Pouget, Jacobo Sitt, Lionel Naccache, Isabelle Arnulf, Delphine Oudiette

**Affiliations:** 1 Sorbonne Université, Institut du Cerveau—Paris Brain Institute—ICM, INSERM, CNRS, Paris, France; 2 AP-HP, Hôpital Pitié-Salpêtrière, Service des Pathologies du Sommeil, National Reference Centre for Narcolepsy, Paris, France; 3 Sorbonne Université, INSERM, Neurophysiologie Respiratoire Expérimentale et Clinique, Paris, France; 4 AP-HP, Hôpital Pitié-Salpêtrière, Service de Neurophysiologie Clinique, Paris, France

**Keywords:** Consciousness, Sleep

## Abstract

Sleep has long been considered as a state of behavioral disconnection from the environment, without reactivity to external stimuli. Here we questioned this ‘sleep disconnection’ dogma by directly investigating behavioral responsiveness in 49 napping participants (27 with narcolepsy and 22 healthy volunteers) engaged in a lexical decision task. Participants were instructed to frown or smile depending on the stimulus type. We found accurate behavioral responses, visible via contractions of the corrugator or zygomatic muscles, in most sleep stages in both groups (except slow-wave sleep in healthy volunteers). Across sleep stages, responses occurred more frequently when stimuli were presented during high cognitive states than during low cognitive states, as indexed by prestimulus electroencephalography. Our findings suggest that transient windows of reactivity to external stimuli exist during bona fide sleep, even in healthy individuals. Such windows of reactivity could pave the way for real-time communication with sleepers to probe sleep-related mental and cognitive processes.

## Abstract (web summary)

We typically assume that we lose the ability to react to the outside world when sleeping. Oudiette et al. show that, in most sleep stages, humans can use their facial muscles to respond to spoken words during transient ‘connected’ periods.

## Main

Sleep has classically been considered a state in which we cannot react to external stimuli. However, congruent evidence from event-related potentials (ERPs)^1–3^ and intracranial recordings^4,5^ has shown that at least low-level sensory processing is preserved across sleep stages. Further studies indicated that sleepers can even process symbolic stimuli at different cognitive levels of representation, including semantic and decisional stages^6–9^. Learning-related sensory cues presented during sleep positively impact subsequent recall of cue-related material upon awakening^10–12^. Moreover, new associations can be learned during sleep^13^ and can even influence participants’ behavior (for example, smoking reduction) a week later^14^. Recent studies suggest that word-association learning during sleep is possible^15,16^ and could generalize into wakefulness in a cross-modal manner^17^. While all these examples of sensory processing during sleep are thought to occur automatically and unconsciously^4^, some studies have shown an incorporation of sensory stimuli into reported dream content^18,19^, suggesting that, at least sometimes, external stimuli could be processed up to conscious level during sleep. However, the lack of single-trial evidence of stimulus integration during sleep complicates the exploration of the neurophysiological basis of this complex and variable phenomenon. Obtaining behavioral responses that serve as real-time indicators of subjective reports could enable us to analyze brain dynamics associated with sensory and cognitive integration in a trial-by-trial manner.

Because behavioral responses have long been assumed to be possible only during wakefulness, they are either rejected from the analysis^8,20^ or not collected at all in sleep studies. The rare studies that measured behavioral responses in sleeping participants discovered manual behavioral responses during N1 sleep (sleep onset)^3,21–24^, but not in deeper sleep stages. However, the loss of limb muscle tone could mask behavioral responses during consolidated sleep. Facial muscles, which are less affected by muscle atonia than the limbs^25^, could be more suited for assessing behavioral responsiveness. For example, eye movements persist during rapid eye movement (REM) sleep and can be used to signal lucidity in people who are aware of dreaming while asleep^19,26^ (that is, lucid dreamers). Combining eye movements and zygomatic/corrugator contractions, we showed that lucid dreamers could respond to queries sent during their dreams in polysomnography (PSG)-verified REM sleep^19^.

In the present work, we capitalized on this research strategy to further question the behavioral disconnection dogma in sleep and to explore stimuli integration at the behavioral and neurophysiological levels. Based on our previous results^19^, we reckoned that such responsiveness would most likely occur during lucid dreaming. We aimed to first assess behavioral responses during lucid REM sleep and then test whether these results could extend to nonlucid REM sleep and other sleep stages. We recruited 27 participants with narcolepsy, who present excessive daytime sleepiness, a short REM sleep latency and a high frequency of lucid dreams^27^, making them an ideal population to collect multiple lucid dreams in the laboratory^28^. We additionally recruited 21 healthy participants (HP; nonlucid dreamers). Participants were explicitly instructed to perform an auditory lexical decision task while napping by frowning or smiling three times depending on the stimulus type (word versus pseudo-word). Facial electromyography (EMG) on corrugator and zygomatic muscles was recorded in addition to usual polysomnography signals.

We discovered that behavioral responses were possible across most sleep stages, including N2 sleep and nonlucid REM sleep, in both participants with narcolepsy and participants without narcolepsy. Regardless of the group or sleep/wake stages, responsiveness was associated with previously validated electrophysiological markers of higher cognitive states. Finally, we found electrophysiological and subjective (postnap reports) evidence for conscious processing of external stimuli during lucid REM sleep. Our findings demonstrate that sleepers can transiently process external stimuli at a high cognitive level and behaviorally respond to them across most sleep stages.

## Results

### Sleeping participants can respond to auditory stimuli

**Fig. 1:**
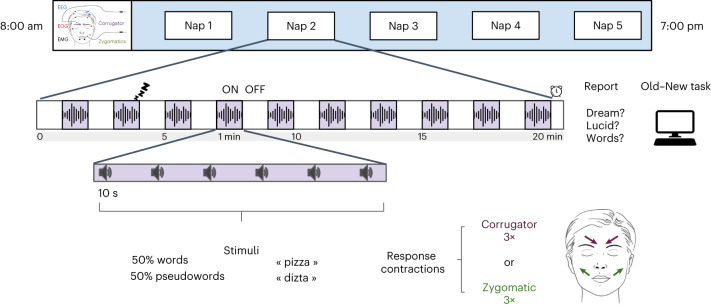
Experimental design. Participants with narcolepsy went through five 20-min naps during the same day. In each nap, periods with stimulation (ON) alternated, every minute, with periods when no stimulus was presented (OFF). During the ON periods, participants were presented with words and pseudo-words and asked to either frown (corrugator muscle contractions) or smile three times (zygomatic muscle contractions) in response to the stimuli. Stimuli were presented every 10 s (±1 s). Following each nap, participants were asked to report whether (1) they had any dream, (2) they were lucid and (3) they recalled any words presented during the nap. Immediately after this debriefing, participants performed a forced-choice ‘old/new’ recognition task. Healthy participants went through the exact same procedure except that they had a single 100-min nap.

**Fig. 2:**
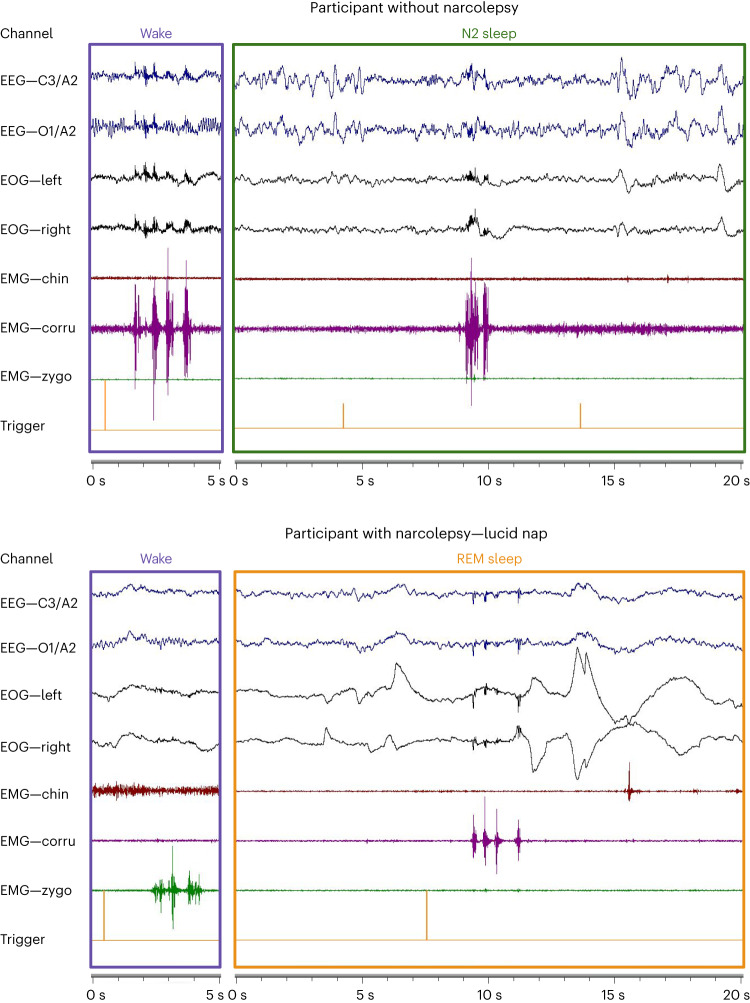
Examples of behavioral responses during N2 sleep in healthy participants (top) and during lucid REM sleep in participants with narcolepsy (bottom). Wake periods corresponding to the same participants are shown on the left side of the figures as a comparison. The orange vertical line on the last channel indicates the stimulus onset. In these examples, we observed the typical markers of N2 sleep: spindles (EEG); and REM sleep: low chin tone (EMG), rapid eye movements (EOG) and θ rhythm (EEG).

**Extended Data Fig. 1:**
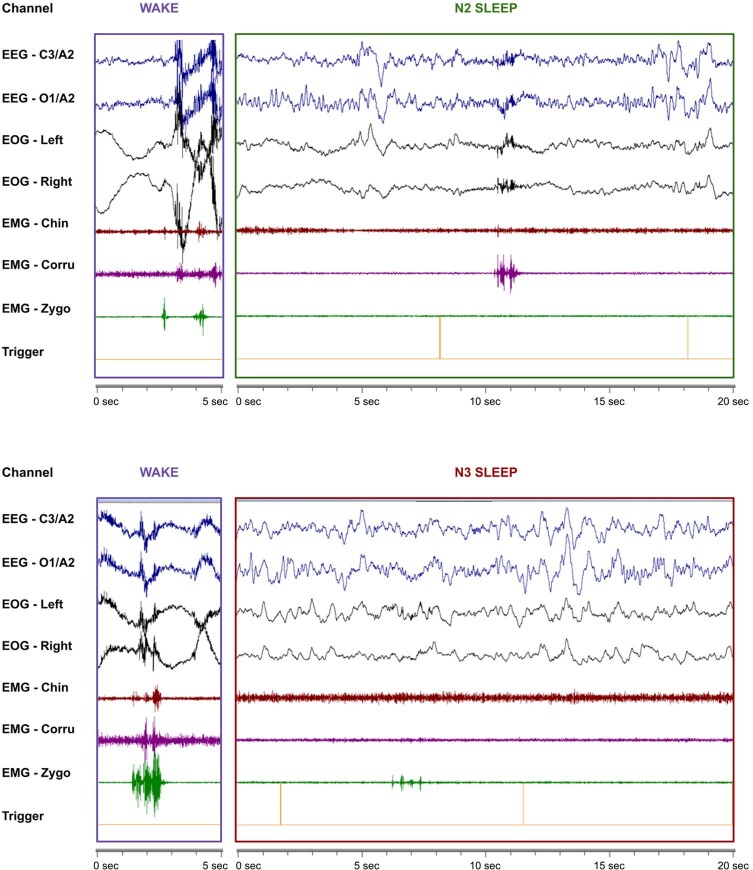
Examples of response during N2 (upper panel) and during N3 sleep (lower panel) in participants with narcolepsy. Wake periods corresponding to the same participants are shown on the left side of the figures. The orange vertical line on the last channel indicates the stimulus onset. Responses to stimuli corresponded to contractions of the zygomatic or corrugator muscles. All raw EEG and behavioral data are available on OSF (see Data Availability statement).

**Extended Data Fig. 2:**
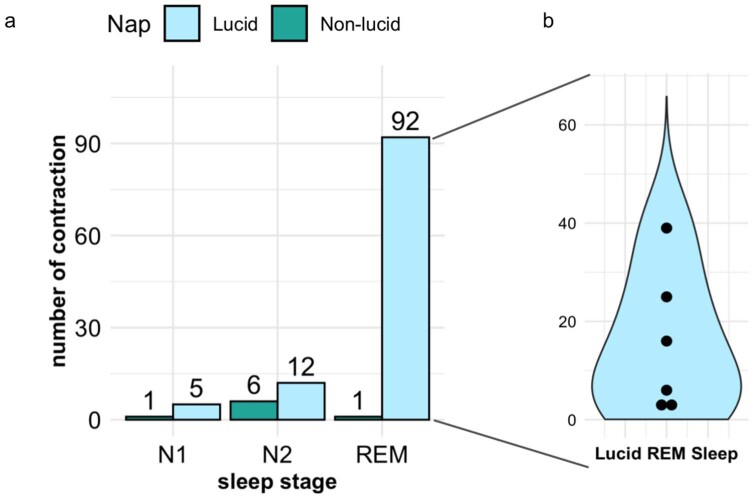
Mixed contractions to signal lucidity. (**a**) Number of mixed contractions (objective lucidity code) in different sleep stages during naps that participants reported to be lucid (sky blue) or non-lucid (green). The relative number of lucidity codes can be found on top of the bars. (**b**) Number of lucidity codes exhibited by each participant in lucid REM sleep. We did not observe any mixed contractions in participants without narcolepsy (HP). On the other hand, we observed a total of 117 mixed contractions from 12 participants with narcolepsy (NP) in 19 different naps. Importantly, all 19 naps contained responses to the stimuli during N2 and/or REM sleep. Among the 117 mixed contractions, 93 were observed in REM sleep, 92 being in naps that were reported (upon awakening) to be lucid (Supplementary Fig. S7). Moreover, 18 contractions were observed in N2 sleep (12 being in lucid naps) and 6 contractions were observed in N1 sleep (5 being in lucid naps).

In this study, we tested participants’ ability to behaviorally respond to auditory verbal stimuli across different sleep stages. We included both participants with narcolepsy (NP, *n* = 27) and HP (*n* = 21). Their sleep/wake stage was continuously monitored by polysomnography (electroencephalography (EEG), electrooculography (EOG) and EMG). Words and pseudowords were verbally presented in a pseudorandomized order during daytime naps; 1-min periods of stimulation (ON periods) alternated with 1-min periods without stimuli (OFF periods; Fig. 1). Participants were instructed to perform a lexical decision task by frowning or smiling three times according to the stimulus type (behavior–stimulus matching was counterbalanced across participants) every time they heard a stimulus, whether they were awake or asleep. As we previously showed^19^, such behavioral responses are visible on surface EMG sensors measuring corrugator (frowning) and zygomatic (smiling) isometric contractions (see Fig. 2, Extended Data Fig. 1 and Supplementary Figs. 1–11 for examples). At the end of each nap, participants reported the following: (1) their mental content during the nap, (2) whether they had a lucid dream during the nap and (3) whether they recalled having actively performed the lexical task while sleeping. They also underwent an old/new task (Supplementary Results). Each nap was labeled as lucid or nonlucid according to participants’ postnap subjective reports, and all REM-sleep trials from this nap were labeled as lucid or nonlucid accordingly. Participants were also instructed to signal their lucidity (if any) with a ‘mixed code,’ by frowning and then smiling once. These objective dream-lucidity signals typically matched participants’ subjective reports upon awakening (Extended Data Fig. 2).

**Fig. 3:**
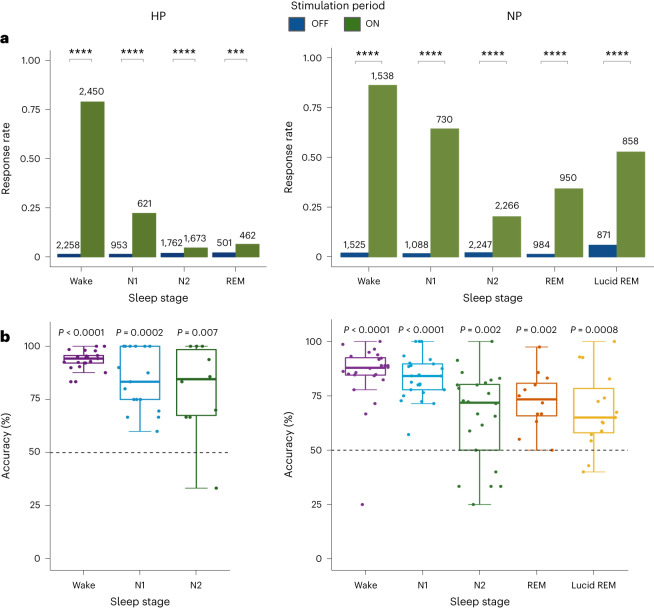
Accurate behavioral responses in both populations. **a**, The overall response rate across different sleep stages during OFF (blue) and ON (green) stimulation periods in participants without (left) and with (right) narcolepsy. The total number of trials in a given condition is indicated on top of the bars. We used binomial generalized mixed-linear models with participants as a random factor for statistical analysis. All *P* values are corrected for multiple comparisons using the Benjamini–Hochberg procedure. Response rates were significantly larger in ON than in OFF periods (pairwise post hoc two-sided comparisons) in HP during wakefulness (*P* < 0.0001), N1 (*P* < 0.0001), N2 (*P* < 0.0001), REM (*P* = 0.0003) and in NP during wakefulness, N1, N2, REM and lucid REM sleep (all *P* < 0.0001). **b**, Accuracy was computed over responsive trials in the lexical decision task for participants without narcolepsy—HP (left) and with narcolepsy—NP (right). Only participants with at least three responses were included in this analysis (number of HP: wake = 21, N1 = 17, N2 = 10; number of NP: wake = 24, N1 = 25, N2 = 24, REM = 12, lucid REM = 15). Each dot represents a participant and dashed lines indicate the 50% chance level. The boundaries of the boxes represent the first and third quartiles (Q1 and Q3, respectively), the midline represents the median and the whiskers depict Q1 − 1.5× IQR and Q3 + 1.5× IQR. One-sided Wilcoxon signed-rank test revealed that both HP and NP were significantly more accurate than chance in all tested sleep stages (corresponding *P* values are indicated in the figure). IQR, interquartile range. ****, *P* < 0.0001; ***, *P* < 0.001. Source data

**Extended Data Fig. 3:**
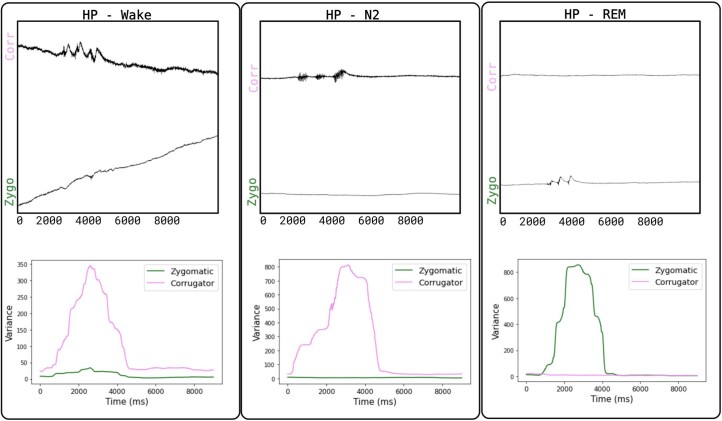
Automatic detection of the response contractions. Upper panel: Examples of EMG traces showing corrugator (pink) and zygomatic (green) muscles contractions in Wake, N2 and REM sleep in healthy participants (HP). Lower panel: EMG variance modulations computed by the response detection algorithm in the corresponding trials. EMG variance drastically increases in the very time when contractions are visible on the EMG signal and only for the contracted muscle. Note that this method is robust to the slow drifts in the EMG signal (as shown in Wake, left panel) and only detects sudden modulations in the signal such as muscle contractions.

**Extended Data Fig. 4:**
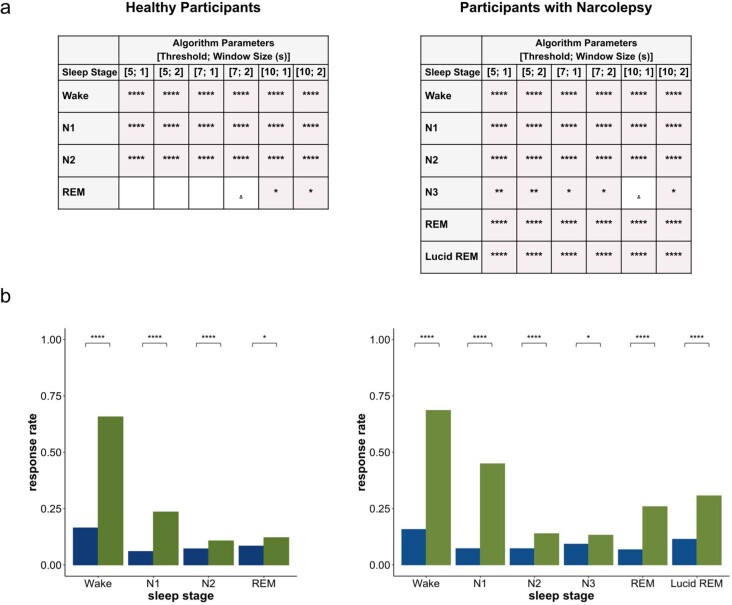
Automatic response detection algorithm detects significantly more contractions during ON stimulation periods compared to OFF stimulation periods in different sleep stages and mirrors the manual scoring. (**a**) Statistical significance of differences in contraction rates between ON and OFF stimulation periods in healthy participants (left) and participants with narcolepsy (right) found by the algorithm using different parameter combinations [Threshold *k*: 5, 7, 10; Window size: 1 or 2 seconds]. Significant differences revealed by post-hoc two-sided pairwise comparisons following linear mixed models are indicated (****: p < 0.0001, ***: p < 0.001, **: p < 0.01, *: p < 0.05, dot: p < 0.1. (**b**) Response rates in ON and OFF stimulation periods found by the algorithm using the strictest parameter combination: threshold *k* = 10 and window size = 2 seconds. The algorithm labeled a trial as responsive if the variance of a 2 second window exceeded 10 times the baseline variance. Post hoc two-sided pairwise comparisons revealed significant differences in HP during wake (*z* = 33.82; *p* < 0.0001), N1 (*z* = 9.49; *p* < 0.0001), N2 (*z* = 4.45; *p* < 0.0001) and REM sleep (*z* = 1.98;*p* = 0.048); and in NP during wakefulness (*z* = 26.34; *p* < 0.0001), N1 (*z* = 15.73; *p* < 0.0001), N2 (*z* = 7.27; *p* < 0.0001), N3 (*z* = 2.11; *p* < 0.0001); REM (*z* = 10.16; *p* < 0.0001) and lucid REM sleep (*z* = 9.85; *p* < 0.0001) after correction for multiple comparisons using Benjamini-Hochberg procedure. Please note the similarity between this figure and Fig. 3a, indicating that response rates found by the algorithm followed the same trend as the manual scoring. Source data

**Extended Data Fig. 5:**
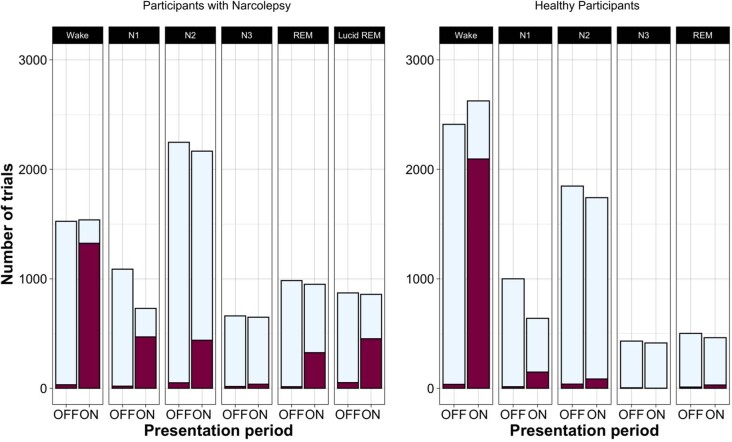
Number of trials during ON and OFF stimulation periods in different sleep stages in participants with (left) and without (right) narcolepsy. The partition of trials containing a response is filled with dark red color in both stimulation periods. Source data

We assessed responsiveness to task stimuli across sleep stages in the two groups, by visually inspecting the corrugator and zygomatic EMG while blind to the sleep stage and the presence/absence of a stimulus. Results from our visual scoring were consistent with those provided by an automatic algorithm (Supplementary Note and Extended Data Figs. 3 and 4) We compared response rates (including both correct and incorrect responses from the two muscles) during ON and OFF stimulation periods (Fig. 3a and Extended Data Fig. 5). Notably, we excluded all responses performed during micro-arousals, keeping only periods when participants were asleep according to the sleep scoring rules^29^. As expected, we found significantly higher response rates during ON versus OFF periods, both during wakefulness (HP: 78.8% versus 1.5%, *z* = 30.02, *P* < 0.0001; NP: 86.1% versus 2.1%, *z* = 27.02, *P* < 0.0001, after false discovery rate (FDR) correction) and N1 sleep (HP: 22.2% versus 1.5%, *z* = 10.99, *P* < 0.0001; NP: 64.2% versus 1.7%, *z* = 18.29, *P* < 0.0001) in both groups. Crucially, we also found, in both HP and NP, significantly higher response rates in ON versus OFF periods during N2 (HP: 4.7% versus 1.9%, *z* = 4.52, *P* < 0.0001; NP: 20.27% versus 2.2%, *z* = 16.57, *P* < 0.0001) and (nonlucid) REM sleep (HP: 6.5% versus 2.2%, *z* = 3.59, *P* = 0.0003; NP: 34.2% versus 1.4%, *z* = 13.93, *P* < 0.0001). We did not find a significant difference between ON and OFF periods in HP during N3 sleep (0.2% versus 0.9%, *z* = −1.23, *P* = 0.22), but we found significantly more responses during ON than OFF periods in N3 sleep in participants with narcolepsy (5.7% versus 2.4%, *z* = 3.31, *P* = 0.0009). Note that the response rates were higher in NP than in HP during ON stimulation periods in all sleep stages (N1: *z* = 4.74, *P* < 0.0001; N2: *z* = 4.44, *P* < 0.0001; N3 sleep: *z* = 3.66, *P* = 0.0002; REM: *z* = 4.95, *P* < 0.0001). This was not true for OFF stimulation periods, during which the two groups had similar contraction rates (*χ*²(1) = 0.03, *P* = 0.87) in all sleep stages (no interaction). Response rates during ON periods decreased significantly from wake to N1 sleep, REM sleep and then N2 sleep (in order) in both HP and NP (Fig. 3a and Supplementary Table 1). Thus, participants could provide behavioral motor codes during most sleep stages, but response frequency decreased in function of sleep depth. Interestingly, only NP reported having performed the task during sleep upon awakening.

To verify that participants actually performed a lexical decision while asleep, we next computed participant-level accuracy scores (Fig. 3b and Supplementary Fig. 12). Note that we did not have enough responsive trials per participant to perform this analysis in REM sleep in HP. Both HP and NP performed the task significantly more accurately than the chance level in all tested sleep stages, with median accuracy above 71% (HP: wake 94.2%, *P* < 0.0001; N1 83.3%, *P* = 0.0002; N2 84.5%, *P* = 0.007. NP: wake 87.9%, *P* < 0.0001; N1 84.1%, *P* < 0.0001; N2 71.8%, *P* = 0.002; nonlucid REM sleep 73.37%, *P* = 0.002). We observed a significant main effect of the sleep stages on accuracy in both HP (*χ*²(2) = 11.01, *P* = 0.004) and NP (*χ*²(4) = 38.23, *P* < 0.0001), indicating a decrease in performance from wake to deeper sleep stages. Moreover, accuracy was positively correlated with increased responsiveness (Supplementary Results). Interestingly, accuracy was higher in HP than in NP (*χ*²(1) = 13.65, *P* = 0.0002) in all tested sleep stages.

**Fig. 4:**
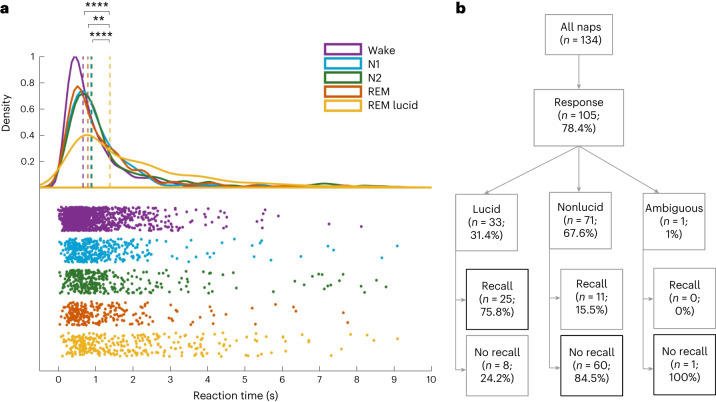
Lucidity is associated with longer reaction times and increased responsiveness. **a**, Distribution of reaction times from stimulus onset to response in correct trials (words and pseudowords) in NP across sleep stages. Dashed lines indicate medians. A mixed-linear model with participant as a random factor and pairwise post hoc two-sided comparisons revealed slower reaction times in lucid REM sleep compared to N1 (*P* < 0.0001), N2 (*P* = 0.0001) and REM sleep (*P* = 0.002). *P* values are corrected for multiple comparisons using the Benjamini–Hochberg procedure. **b**, Flowchart detailing the repartition of naps in participants with narcolepsy—the percentage of naps with at least one behavioral response is indicated and the responsive naps are further divided depending on whether participants reported a lucid dream upon awakening and whether they explicitly recalled responding during the nap. ****, *P* < 0.0001; ***, *P* < 0.001. Source data

We then wondered if one behavioral hallmark of lexical decision task during wakefulness—slower response times (RTs) for pseudowords than for words^30^—persisted in our sleeping participants. Only correct responses were included in this analysis. For both NP and HP, we found a main effect of both sleep stages (HP: *χ*²(2) = 25.47, *P* < 0.0001; NP: *χ*²(4) = 82.5, *P* < 0.0001) and stimulus type (HP: *χ*²(1) = 45.59, *P* < 0.0001; NP: *χ*²(1) = 36.9, *P* < 0.0001) on RTs; crucially, there was no significant interaction effect between these two factors (HP: *χ*²(2) = 2.7, *P* = 0.25; NP: *χ*²(4) = 7.3, *P* = 0.1), suggesting that the effect was not modulated by sleep stage (Supplementary Fig. 13). Reponses to pseudowords were on average 100 ms slower than responses to words in HP (median for words: 1.29 s) and 130 ms slower in NP (median for words: 1.42 s). This was also the case for each sleep stage independently. In NP, responses were faster during wakefulness than during sleep (median RT: wake, 1.36 s versus N1 sleep 1.56 s, *P* = 0.034; N2 sleep 1.59 s, *P* = 0.0001; nonlucid REM, 1.49 s, *P* = 0.0001), whereas no significant differences were found between sleep stages (Fig. 4a). A similar pattern was observed for HP, including significantly shorter reaction times in wakefulness and N1 than in N2 sleep (wake versus N2 sleep: *t* = 4.6, *P* < 0.0001; N1 versus N2 sleep: *t* = 2.64, *P* = 0.008). Moreover, we found significantly shorter reaction times in accurate trials compared to inaccurate ones in wake (*t* = −6.91, *P* < 0.0001), N1 sleep (*t* = −2.31, *P* = 0.021) and N2 sleep (*t* = −3.82, *P* = 0.0001) in HP and in wake (*t* = −5.56, *P* < 0.0001), N1 sleep (*t* = −2.24, *P* = 0.025) and REM sleep (*t* = −5.275, *P* < 0.0001) in NP. We also examined the response latencies of the two response types (zygomatic and corrugator contractions) to ensure that they had the same level of difficulty. We found similar response latencies for the two muscles in all sleep stages (no interaction was found with the sleep stage) for both participants with (*t* = 1.38, *P* = 0.18) and without narcolepsy (*t* = 0.88, *P* = 0.38).

We finally assessed whether lucid and nonlucid REM sleep differed on the behavioral and subjective levels. Only NP reported lucid dreams upon awakening, in 33/134 naps (24.6%). Like in nonlucid REM sleep, response rates were higher during ON versus OFF periods in lucid REM sleep (52.7% versus 6%, *z* = 18.04, *P* < 0.0001). Accuracy was also better than chance (65% versus chance level at 50%, *P* = 0.0008, one-sided Wilcoxon signed-rank test) and not statistically different than in nonlucid REM sleep (*t* = 1.24, *P* = 0.3). Reaction times were significantly shorter in accurate trials compared to inaccurate ones (*t* = −2.61 *P* < 0.009). Notably, lucidity significantly increased the response rate in REM sleep (*z* = 7.97, *P* < 0.0001) to a level similar to the one observed in N1 sleep (Fig. 3a and Supplementary Table 1). Interestingly, RT was significantly longer during lucid REM sleep than during wakefulness but also than during other sleep stages (median RT: lucid REM sleep, 2.1 s versus N1 sleep, 1.56 s, *P* < 0.0001; versus N2 sleep, 1.59 s, *P* = 0.0001; versus nonlucid REM sleep, 1.49 s, *P* = 0.002; Fig. 4a). Finally, after naps associated with at least one behavioral response during sleep, participants who reported lucidity recalled more frequently having performed the task during sleep (task recall after 75.8% of lucid naps versus 15.5% of nonlucid naps; *χ*²(2) = 36.15, *P* < 0.0001; Fig. 4b).

### Local brain activations during sleep in responsive trials

Sleep/wake stages were scored according to established guidelines^29^ by a certified sleep expert blind to the responses (corrugator and zygomatic EMG channels were removed for sleep scoring). Figure 2, Extended Data Fig. 1 and Supplementary Figs. 1–11 show 15 examples of responses during bona fide sleep for both HP (in N2 and REM sleep) and NP (in N2, REM and N3 sleep). Given the novelty and importance of this result, we performed additional analyses to confirm that these responsive trials indeed occurred during sleep periods.

**Fig. 5:**
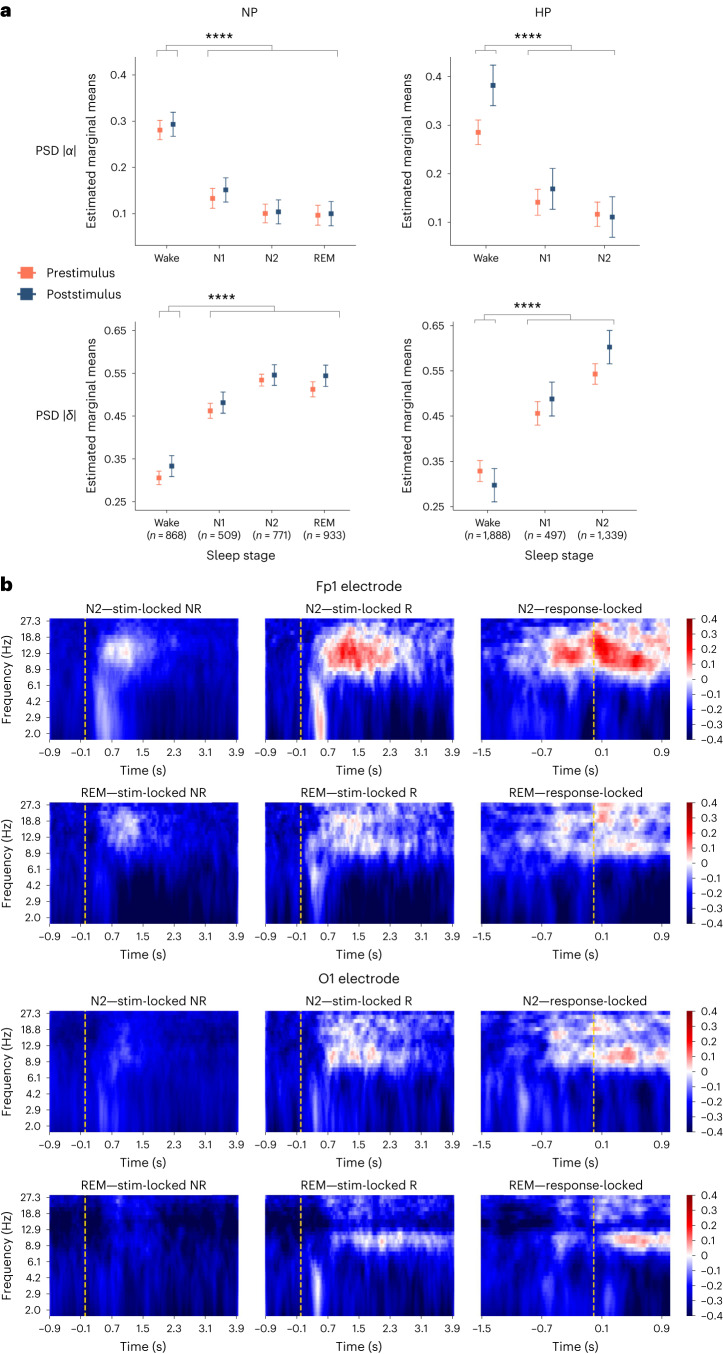
Participants exhibit sleep activity in responsive trials, with local brain activations associated with responsiveness. **a**, Normalized PSD values in α (PSD |*α*|) and δ (PSD |*δ*|) frequencies in responsive trials across different sleep stages in prestimulus and poststimulus periods. Prestimulus marker values are computed over the 1 s-period before the stimulation, whereas poststimulus marker values are calculated in the 8s-period following the stimulation. Data are presented as mean values ± 95% confidence intervals. *n* depicts the number of datapoints included in the statistical analysis, taken from 25 NP (22 in wake, 24 in N1, 23 in N2 and 15 in REM) and 21 HP (21 in wake, 21 in N1 and 20 in N2). Please note that marker values in different sleep stages were never at wake level as revealed by a linear mixed model with participant ID as random effect (****P < 0.0001 in pairwise post hoc two-sided comparisons, adjusted for multiple comparisons), indicating that participants were indeed asleep while they were responding. **b**, Time–frequency analysis (TFA) performed on the Fp1 (top) and the O1 (bottom) electrodes in N2 (23 nonresponsive participants and 21 responsive participants) and REM sleep (15 nonresponsive participants and 14 responsive participants) of NP. Left and middle, stimulus-locked TFA in nonresponsive and responsive trials, respectively. Right, response-locked TFA. Transient and spatially localized increases in α and β frequencies were associated with behavioral responsiveness to the task. Source data

First, we performed spectral analyses during both the baseline period (−1,000 to 0 ms relative to stimulus presentation) and the poststimulus period (0–8,000 ms; Fig. 5a). For both time windows, as well as for all sleep stages in both HP and NP, power spectral densities (PSDs) in responsive trials reflected the expected profile of the given sleep/wake stage. Compared to wake trials, all responsive trials were associated with lower α and higher δ spectral power (see Fig. 5a and Supplementary Table 4 for the statistical comparisons). Additional analyses quantifying classical sleep graphoelements (spindles and slow waves) in responsive and nonresponsive non REM (NREM) sleep are provided in Supplementary Results. Overall, our results are in line with the manual sleep scoring and confirm that the background brain activity in responsive sleep trials presents the typical signatures of sleep.

**Extended Data Fig. 6:**
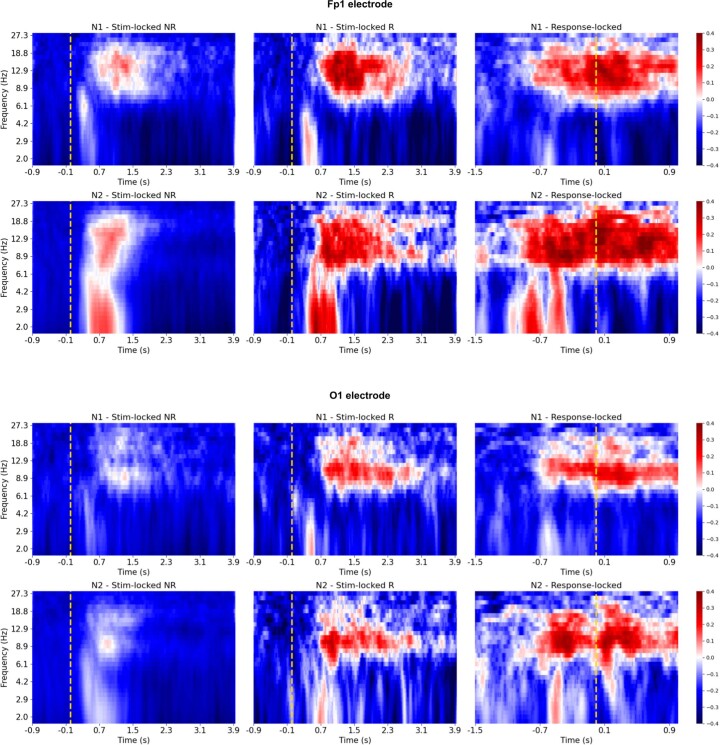
Time-frequency analysis. Time-frequency analysis (TFA) performed on the Fp1 (upper panel) and the O1 (bottom panel) electrodes in N1 and N2 sleep of HP. The Left and middle panels are stimulus-locked TFA in nonresponsive and responsive trials respectively. The right panels show response-locked TFA.

**Extended Data Fig. 7:**
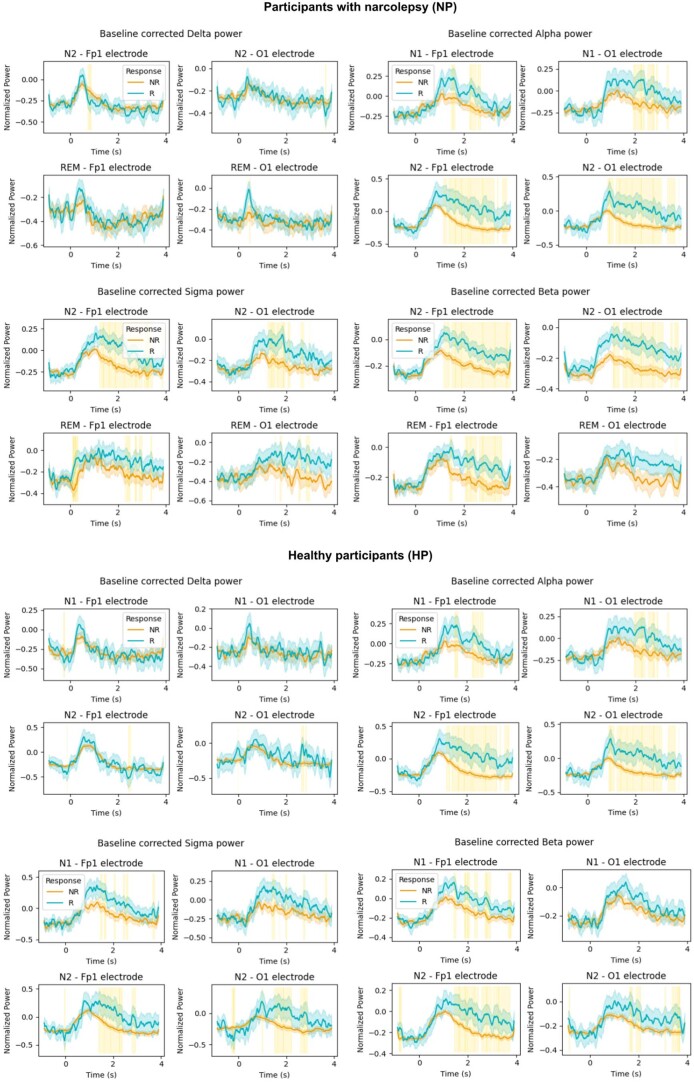
Timing of statistical differences in the time-frequency analysis. Differences in power across time in delta, alpha, low beta/sigma and high beta bands, in responsive (R) and unresponsive (NR) trials during N2 and REM sleep in participants with narcolepsy (upper panel) and during N1 and N2 sleep in healthy participants (lower panel). Time-frequency analysis was performed over Fp1 and O1 electrodes. The error bands depict 95% confidence intervals. Significant differences are indicated by yellow shade (FDR corrected p-value < 0.05, mass-univariate analysis on time dimension using mixed-linear models with responsiveness as the explanatory factor and participant ID as a random effect). Source data

**Extended Data Fig. 8:**
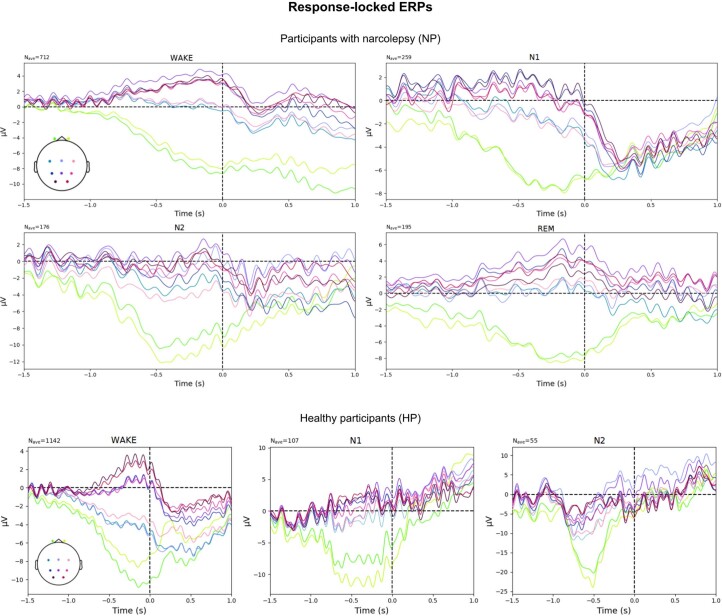
Response-locked ERP analysis in participants with narcolepsy (upper panel) and healthy participants (bottom panel). Dashed vertical lines indicate response start. Please note the motor preparation potential over frontal electrodes in all tested sleep stages.

We next wondered what differences in brain processing could account for the presence or absence of behavioral responses during sleep. We performed a mass-univariate stimulus-locked time–frequency analysis independently for each group and each sleep stage. We applied a log-ratio baseline correction relative to the −1,000 to 0 ms time window to only capture the time–frequency activity induced by stimulus processing. The average time–frequency matrix for responsive and nonresponsive trials is presented in Fig. 5b for NP (N2 and REM sleep) and in Extended Data Fig. 6 for HP (N1 and N2 sleep). This analysis revealed the following: (1) the absence of significant δ-band modulation by responsiveness; (2) compared to nonresponsive trials, a more pronounced and more sustained activation in α (8–12 Hz) and β (12–30 Hz) bands, mostly observed in frontal electrodes, and roughly spanning from 1,200 ms to 3,800 ms poststimulus presentation (see Extended Data Fig. 7 for the exact time-intervals for each frequency band). To assess whether this α- and β-band modulation was due to ultrashort arousals (shorter than the usual 3 s criteria defining micro-arousals) or to cognitive and motor processes induced by the stimuli, we performed a response-locked time–frequency analysis (Fig. 5b, right), as well as response-locked ERPs (Extended Data Fig. 8). We observed a power increase in α- and β-frequency bands starting from 700 ms before the behavioral response; this power increase was predominant on frontal sites and during the period following the response. This spatial profile of activity is different from the typical, occipital activation observed during micro-arousals. Crucially, comparison between the response-locked time–frequency matrix and ERPs revealed that the observed preresponse α and β band activation was concomitant with a motor preparation potential (Bereitschafts potential), observed mainly on frontal electrodes, in all wake/sleep stages in both NP and HP. This frontal location is consistent with the known physiology of facial muscles motor preparation and execution^31–33^.

In sum, our results suggest that participants’ responses happened on a global background of sleep brain activity (with a similar stage-specific physiology compared to nonresponsive trials), but that they involve local (in time and space) brain activations likely linked to cognitive and motor processing of the stimulus. Taken together with our behavioral results, these results demonstrate that sleepers can perceive verbal stimuli, make a lexical decision and perform an adequate motor response while remaining asleep in N1, N2 and REM sleep. The fact that participants’ responses were accurate and slower for pseudowords than for words suggests that stimuli were processed at a high cognitive level (at least beyond the lexical level). These results overall suggest the existence of transient states that allow responsiveness to external information during sleep, whose frequency and duration depend on the sleep stage.

### EEG markers of high cognitive states predict responsiveness

To explore whether responsiveness during sleep could be explained by an ongoing, richer cognitive state before stimulation in nonlucid participants (NP and HP), we computed electrophysiological markers known for distinguishing high versus low cognitive states^34,35^. These markers were previously shown to differentiate patients with unresponsive wakefulness syndrome from patients in a minimally conscious state and HP^34,36,37^, as well as wakefulness and REM sleep from N3 sleep^38^. In addition to classical spectral measures (normalized PSDs in δ, θ, α, β and γ frequency bands), we included one connectivity measure (weighted symbolic mutual information (wSMI) in the θ band), and three complexity measures (the Kolmogorov complexity (KC), the permutation entropy in the theta band (PE θ) and the sample entropy (SE)). Crucially, we computed these markers in the 1,000 ms time window before the stimulus presentation; therefore, these markers reflected the ‘resting-state’ brain dynamics of the participants just before the stimulus presentation, and not the evoked activity of the stimulus or the response.

**Extended Data Fig. 9:**
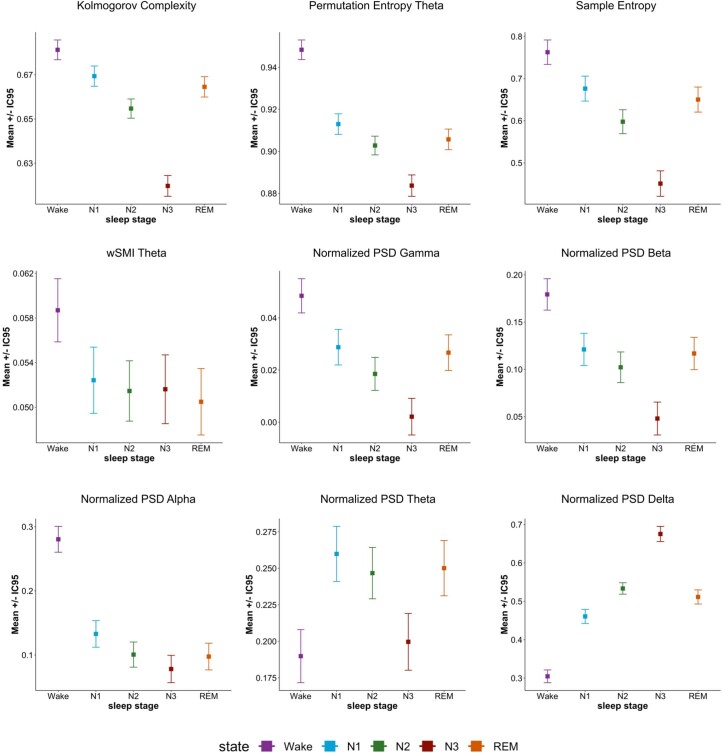
Evolution of electrophysiological markers across sleep stages in participants with narcolepsy (NP). Three complexity measures (the Kolmogorov Complexity -KC, the Permutation Entropy -PE, and the Sample Entropy -SE), one connectivity measure (weighted symbolic mutual information (wSMI) in the theta band), and five spectral measures (normalized power spectral densities (PSD) of delta, theta, alpha, beta and gamma frequency bands) were computed separately for the wake (N = 961 from 22 NP), N1 (N = 505 from 24 NP), N2 (N = 1186 from 23 NP), N3 (N = 435 from 16 NP), and REM (N = 587 from 15 NP) sleep stages in participants with narcolepsy. The results in healthy participants can be found in Supplementary Fig. S3. Each dot indicates marginal means estimated by a mixed-linear model including sleep stage as an independent variable, EEG marker as the dependent variable, and participant ID as a random variable. Error bars depict 95% confidence intervals. Complexity and high-frequency PSD decreased in sleep compared to wake (wake > N1 > REM sleep > N2 > N3), whereas delta PSD increased with sleep (N3 > N2 > REM sleep > N1 > wake). Details of the statistical comparisons can be found in Supplementary Table S5. Source data

**Extended Data Fig. 10:**
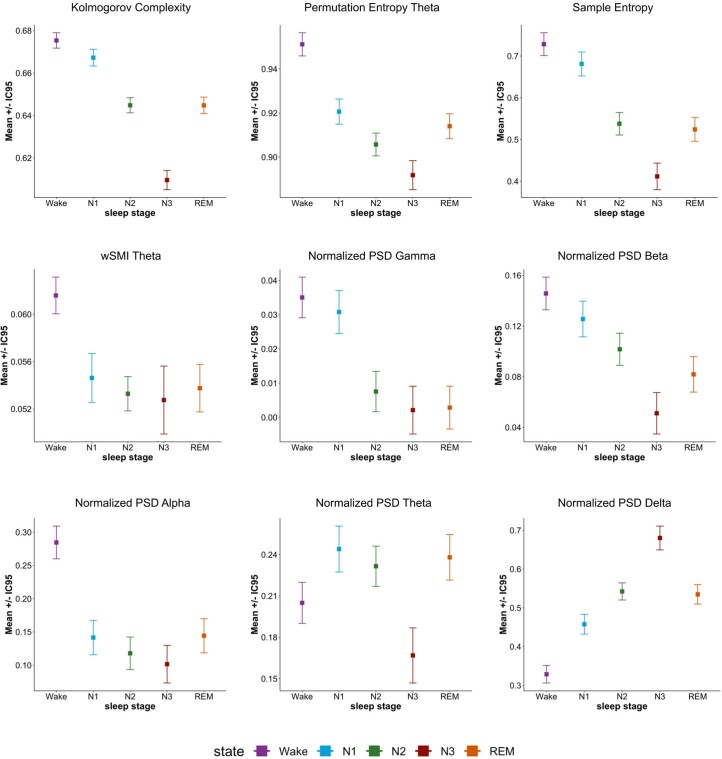
Evolution of electrophysiological markers across sleep stages in healthy participants (HP). Three complexity measures (the Kolmogorov Complexity -KC, the Permutation Entropy -PE, and the Sample Entropy -SE), one connectivity measure (weighted symbolic mutual information (wSMI) in the theta band), and five spectral measures (normalized power spectral densities (PSD) of delta, theta, alpha, beta and gamma frequency bands) were computed separately for the wake (N = 981 from 21 HP), N1 (N = 373 81 from 21 HP), N2 (N = 1339 from 20 HP), N3 (N = 166 from 8 HP), and REM (N = 451 from 10 HP) sleep stages in HP. Each dot indicates marginal means estimated by a mixed-linear model including sleep stage as an independent variable, marker as the dependent variable, and participant ID as a random variable. Error bars denote 95% confidence intervals. Complexity and high-frequency PSD decreased in sleep compared to wake (wake > N1 > N2 ≈ REM > N3), whereas delta PSD increased with sleep (N3 > N2 ≈ REM > N1 > wake). Theta PSD was higher in N1 and lower in N3 sleep. Details of the statistical comparisons can be found in Supplementary Table S6. Source data

To ensure that these markers would provide meaningful information about the cognitive state of our participants, we first assessed how the markers varied in different sleep stages as a sanity check (NP: Extended Data Fig. 9, HP: Extended Data Fig. 10). As expected, we found that complexity, connectivity values and high-frequency PSD decreased from wake to N1 sleep, REM sleep, N2 sleep and N3 sleep (in order), this descending profile mirroring the response rates (see Supplementary Tables 3 and 4 for statistical comparisons between the different sleep stages, for each marker and each group). The reverse was observed for δ PSD. These results demonstrated that our markers can reliably distinguish participants’ sleep/wake stages.

**Fig. 6:**
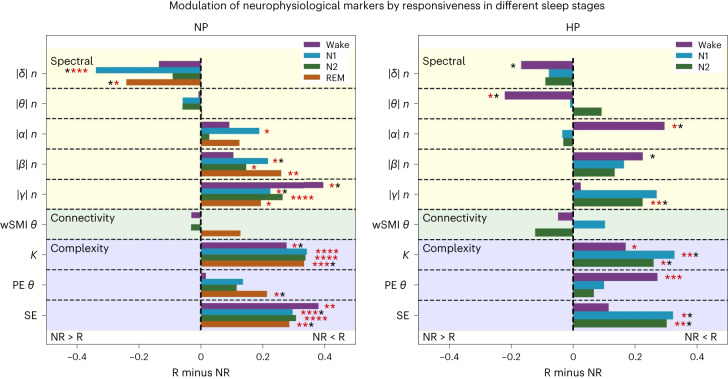
EEG markers of high cognitive states computed before stimulus presentation vary with responsiveness to stimuli. After the *z* score transform of marker values, we subtracted the marginal estimated mean of nonresponsive trials (NR) from responsive (R) trials for each marker and each stage (represented by bars). Statistical comparisons between the responsive and nonresponsive trials were made using linear mixed models with participant ID as random effect. Asterisks represent statistical significance in pairwise post hoc two-sided comparisons. ****, *P* < 0.0001; ***, *P* < 0.001; **, *P* < 0.01; *P* < 0.05; red stars indicate significance after FDR correction for 72 comparisons. All adjusted *P* values and averaged marker values can be found in Supplementary Tables 7 and 8. Almost all markers showed a variation in the direction corresponding to increased cognitive states when contrasting responsive trials to nonresponsive trials (for example, increased EEG complexity and decreased EEG δ power), both in participants with (left) and without narcolepsy (right). Note the similarity with Fig. 3 in ref. ^33^ that contrasted conscious to nonconscious states in patients suffering from disorders of consciousness. R, responsive; NR, nonresponsive. Source data

Next, we assessed how these electrophysiological markers differed in responsive and nonresponsive trials, except during REM sleep in HP (not enough remaining responsive trials after EEG preprocessing). Figure 6 shows the difference in the estimated marginal means of the *z*-scored marker values in responsive and nonresponsive trials for each sleep stage in nonlucid NP (left) and HP (right; see Supplementary Tables 7 and 8 for detailed comparisons). Positive marker values indicate an increase of the markers in the responsive trials compared to nonresponsive trials, whereas negative marker values signify a decrease in the responsive trials. Our analysis revealed similar patterns of variations in nonlucid NP and HP, including an increase in the EEG complexity and in the high-frequency PSD, and a decrease in the δ PSD in responsive trials versus nonresponsive trials. Connectivity (wSMI) did not differ in the two conditions.

**Fig. 7:**
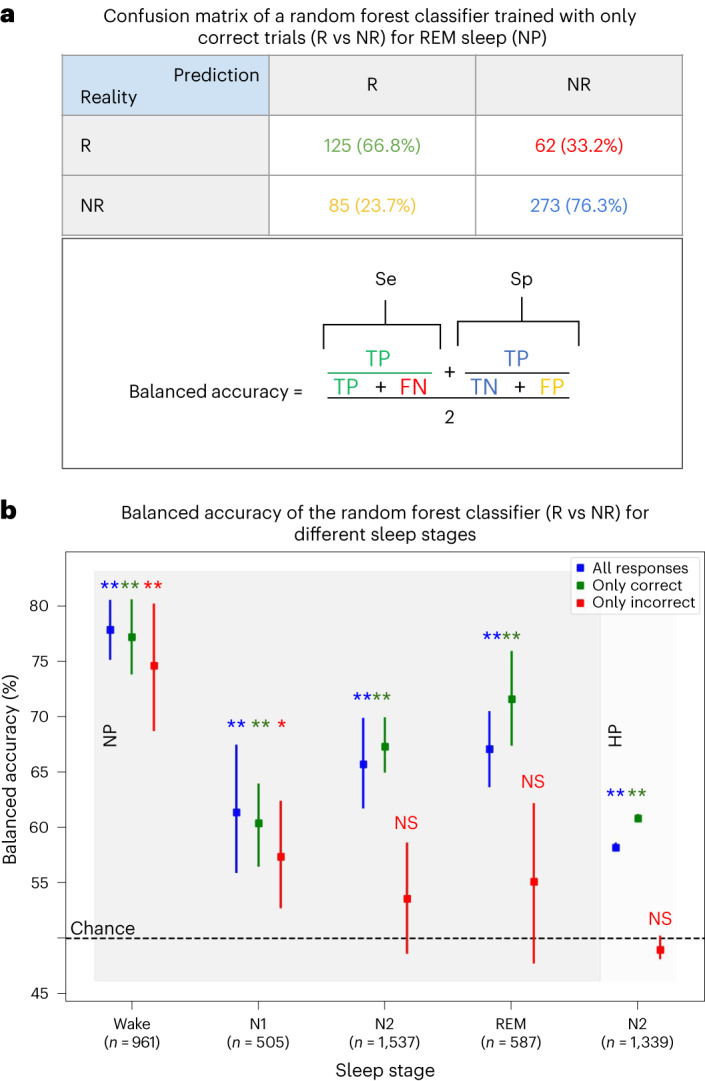
EEG markers of high cognitive states computed before stimulus presentation predict responsiveness to stimuli in each nonlucid sleep stage. **a**,**b**, We fed a random forest classifier with our nine EEG markers and trained it to classify R trials versus NR ones using a tenfold cross-validation method. We conducted this analysis considering all responses (in blue), then separately for both correct (in green) and incorrect responses (in red). A confusion matrix for correct REM sleep trials in nonlucid naps of participants with narcolepsy is shown in **a**, with a description of the balanced accuracy measure that we computed to take unbalanced datasets into account. The confusion matrix for each stage and group can be found in Supplementary Table 9. Balanced accuracy scores are plotted in **b** for different sleep stages, in function of response accuracy, both for participants with narcolepsy (wake, N1, N2, REM sleep; left) and without narcolepsy (N2, right), with the corresponding statistical significance against chance level computed with a 500 permutations test (all trials: all NP *P* values = 0.002, HP *P* value = 0.006; correct trials: all *P* values = 0.002; incorrect trials: wake *P* = 0.002, N1 *P* = 0.04. Note that 0.002 is the smallest *P* value obtainable via 500 permutations). Data are presented as mean values ± 95% confidence intervals. *n* represents a number of datapoints in all (correct + incorrect) trials, taken from 22 NP in wake, from 24 NP in N1, from 23 NP in N2, from 15 NP in REM and from 20 HP. TP, responsive trials classified as responsive; TN, nonresponsive trials classified as nonresponsive; FP, false positives (NR trials classified as responsive). FN, false negatives (R trials classified as nonresponsive). Source data

To further explore the predictive power of these EEG markers on responsiveness, we trained a random forest classifier using a multivariate combination of these markers collected in nonlucid NP and did so independently for each sleep stage. We then tested whether this classifier could predict responsiveness on a trial-by-trial basis in both NP (using a classical stratified cross-validation procedure) and HP trials (in N2 sleep). The balanced accuracy score was above 60% for all sleep stages in NP nonlucid naps (reaching 67% for REM sleep) and reached 58% for N2 sleep in HP (Fig. 7). All balanced accuracy scores were significantly different than the chance level computed by a 500-permutation procedure (*P* < 0.002 for all stages in NP, and *P* = 0.006 for N2 sleep in HP), with a mean balanced accuracy score of permutation trials around 50% for all stages (Supplementary Table 9).

One could argue that these EEG markers measure differences in motor capacities (that is, more or less motor inhibition) rather than differences in cognitive capacities. To explore this possibility, we tested whether our classifier better predicted responsiveness when including only correct responses. Prediction performance increased for all sleep stages in both NP and HP (except for N1 in NP where performance slightly decreased while remaining significantly higher than chance; Fig. 7b). Balanced accuracy reached 72% for REM sleep in NP and 61% for N2 sleep in HP (*P* = 0.002, 500-permutation procedure). Next, we tested the opposite by including only incorrect responses. Interestingly, prediction performance drastically decreased, falling to chance level for all sleep stages other than N1 sleep in NP (where it also decreased significantly while remaining higher than chance level; Fig. 7c). The fact that prediction performance was driven by correct responses is a strong indicator that the differences in brain dynamics measured by the EEG markers reflected differences in cognitive processing between responsive and nonresponsive trials, and not mere motor capacities.

In sum, our EEG results suggested that a particular brain state before the stimulation, characterized by increased complexity and faster oscillations, allowed responsiveness during sleep. A multivariate combination of these markers predicted the presence/absence of response in a trial-by-trial level. The facts that (1) the markers varied with responsiveness similarly in nonlucid NP and HP and (2) the classifier trained with NP data could classify responsive trials in HP better than chance strongly suggest that the same brain dynamics underlie responsiveness in both participants with and without narcolepsy (in nonlucid sleep). Finally, the finding that the predictive power of those markers was driven by correct trials strongly suggests that behavioral responses were due to higher cognitive processing of the stimulus.

### Conscious processing of external stimuli in lucid dreaming

**Fig. 8:**
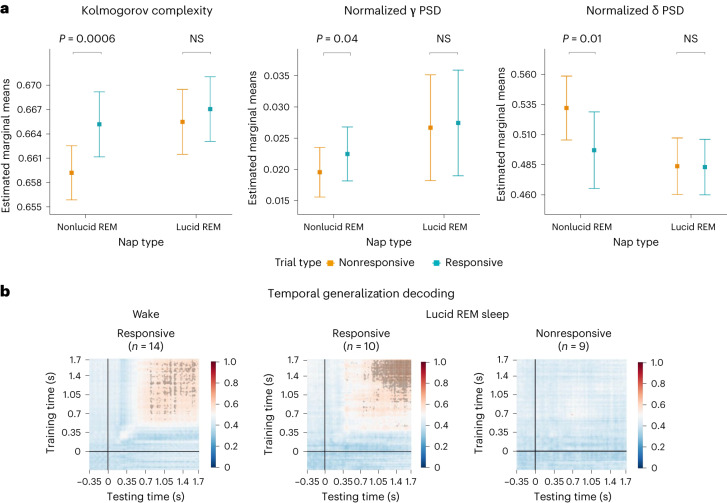
Effect of lucidity on EEG markers and response to stimuli in participants with narcolepsy. **a**, Top, Kolmogorov complexity (left), normalized γ PSD (norm-γ; middle) and normalized δ PSD (norm-δ; right) before stimuli onset as a function of whether the stimulus will be followed by a behavioral response (in blue) or not (in orange), for lucid and nonlucid REM sleep in participants with narcolepsy. Data are presented as mean values ± 95% confidence intervals. Statistical differences computed via linear mixed models, adjusted for multiple comparisons are indicated (NS, nonsignificant). A number of datapoints in the model are 229 (from 13 participants) for responsive REM sleep, 358 (from 15 participants) for nonresponsive REM sleep, 353 (from 15 participants) for responsive lucid REM sleep and 333 (from 16 participants) for nonresponsive lucid REM sleep. Kolmogorov complexity and norm-γ were significantly higher for responsive trials compared to nonresponsive trials in nonlucid naps for all participants. Conversely, the norm-δ was significantly lower in responsive trials in nonlucid naps. No such differences were observed in lucid naps, suggesting a ceiling effect for markers of high cognitive states in lucid naps (see Supplementary Table 10 for statistical details). Overall, Kolmogorov complexity and norm-γ were higher, and norm-δ was lower in lucid naps compared to nonlucid naps irrespectively of the responsiveness. **b**, Time-generalization decoding of stimulus-related brain activity compared to baseline brain activity, in trials with (top) and without (bottom) response, in wake (left) and lucid REM sleep (right). The logistic regression classifier was trained on each time point and then tested on all the time points to obtain a generalization pattern. Each intersection point of a training time and a testing time shows the AUC of the classifier. Time points with an AUC > 0.5 and that are statistically significant are outlined in black (two-sided nonparametric sign test across participants with FDR correction for 41,616 comparisons, *P* < 0.05). Source data

To investigate the specificities of lucid REM sleep in NP, we first compared the electrophysiological markers between responsive and nonresponsive trials in this condition. Interestingly, none of these markers differentiated responsive from nonresponsive trials in lucid REM sleep (all uncorrected *P* > 0.05; Fig. 8a and Supplementary Table 8). Using a Bayesian analysis, we confirmed a true absence of difference between responsive and nonresponsive trials in lucid REM sleep; for each marker, the Bayes factor comparing our full mixed-linear model to the one of a ‘null model’ with only the random effect ranged from 0.21 to 0.08, indicating moderate (<0.33) to strong (<0.1) evidence for the null model^39^.

We next investigated how the marker values in lucid REM sleep differed from the ones in nonlucid (ordinary) REM sleep. Lucid trials were associated with higher complexity (SE), higher normalized PSD of γ and lower normalized PSD of δ values compared to nonlucid trials. Statistical analyses (both frequentist and Bayesian) restricted to responsive trials revealed similar values in lucid and nonlucid conditions for all markers, indicating comparable brain activity during responsive trials between nonlucid and lucid REM sleep (Fig. 8a and Supplementary Table 11).

In sum, lucid REM sleep was characterized by a systematic increase in EEG markers of higher cognitive states, irrespective of behavioral responsiveness to the task, with a pattern of markers similar to the one observed in nonlucid/responsive trials (that is, faster oscillations and higher complexity compared to nonlucid/nonresponsive REM trials). This suggests a ceiling effect for marker values in lucid REM sleep, indicating a sustained high cognitive state during this condition.

This neurophysiological profile combined with the subjective report of having performed the task during sleep (Fig. 4) suggests that NP consciously processed the stimuli when in lucid REM sleep. Several signatures of conscious processing have been described in the literature, including the late P3b component in evoked related potentials^40–42^ or the square-like shape pattern in the temporal generalization method^43,44^ (such a pattern reflects a late, stable and sustained processing stage that has been previously related to conscious access^43–45^) Given our unbalanced dataset, we primarily used the temporal generalization approach to explore consciousness of external stimuli in lucid REM sleep (see Supplementary Fig. 14 for stimulus-locked ERPs). Briefly, this analysis tests how stimulus-induced brain activity differs from baseline activity; it consists in training a linear classifier at each time point to differentiate stimulus-present versus stimulus-absent epochs and testing its performance for all the other time points (for example, training the classifier at *t* = 2 and testing its ability to correctly classify at *t* = 1,2,3,4,5,…, obtaining thus a whole matrix of performance for each training time point/testing time point). We found that responsive trials during lucid REM sleep were associated with the expected square-like shape pattern starting from 350 ms poststimulus presentation (Fig. 8b). This pattern was similar to the one observed in responsive wake trials, indirectly supporting our hypothesis that NP are conscious of the stimuli presented during responsive trials in lucid REM sleep. In contrast, we did not find any discernible decoding pattern for nonresponsive trials in lucid REM sleep, suggesting that NP are not conscious of external stimuli when they do not respond. This result might seem at odds with our previous observation that marker values computed before the stimulation were similarly high in responsive and nonresponsive trials (Fig. 8a) in lucid NP. It suggests that high marker values are indicative of a rich cognitive state, which is permissive (but not necessarily sufficient) for responsiveness during sleep.

Lucid REM sleep trials were therefore associated with (1) a subjective report of having performed the task while sleeping; (2) a systematic increase in EEG markers of higher cognitive states; (3) an electrophysiological signature of conscious processing of external stimuli in responsive trials (temporal generalization pattern) and (4) longer reaction times, suggesting that participants were engaged in a dual task during which external information (outside world, including verbal stimuli) and internal information (ongoing dream) competed for attention^9^. All these findings hint that lucid participants could consciously integrate and respond to external stimuli during sleep.

## Discussion

Our results provide compelling evidence that sleeping humans present transient windows of sensory connection with the outside world during which they process external information at a high cognitive level and can physically respond. Until now, behavioral responsiveness had only been demonstrated during the sleep onset period^3,21,22^ or in the unique case of lucid REM sleep^19^. Our findings go further by demonstrating the possibility of behavioral responsiveness to external stimuli in bona fide sleep in a large group of participants. Responses were associated with temporally and spatially localized activations in the sleeping brain. Although remaining rare in HP, we argue that the existence of these transient windows of behavioral reactivity provides a much more complex picture of sleep/wake phenomena than previously considered. Furthermore, we show that these transient windows of cognitive and behavioral connection are associated with specific brain dynamics (faster oscillatory activity and higher signal complexity), which predict responsiveness on a trial-by-trial basis. Finally, for the particular case of lucid REM sleep, we provide strong arguments in favor of conscious processing of external information, including the presence of a neural signature of conscious access^43^ in responsive trials and explicit recall of having performed the task during sleep.

Our study presents several limitations. First, we primarily chose to assess behavioral responses using a visual inspection of the corrugator and zygomatic muscle activity on EMG channels. We favored this method to an automated algorithm, which requires choosing an arbitrary threshold and would need to be validated against a gold standard (which does not exist). Note however that our visual scoring was consistent with an automatic detection of responses based on EMG signal variance (Supplementary Note and Extended Data Figs. 3 and 4). Second, we only investigated responsiveness during daytime naps, thus preventing us from fully assessing behavioral responsiveness during N3 sleep (not enough trials) and more generally during night-time sleep. Third, our ten-electrodes montage with a mastoid reference was not ideal for computing the wSMI connectivity measure^46^, making the results of this analysis difficult to interpret. Fourth, we used postnap subjective reports to determine lucidity instead of the gold standard, objective signal of lucidity^26,28,47^. Nevertheless, we also collected an objective lucidity signal (successive corrugator and zygomatic contractions) that substantially matched participants’ subjective reports upon awakening, confirming the reliability of subjective reports in determining participants’ lucidity. Finally, we only obtained lucid naps in patients with narcolepsy. Therefore, our results for lucid REM sleep need confirmation in lucid HP.

Although the response rate was minimal during OFF periods (compared to ON periods), it was still greater than zero, which might appear surprising in the absence of stimuli. This can be due to the following factors: (1) participants might have had spontaneous contractions, (2) they might have dreamt about the task and contracted their muscles in response to a dreamt auditory stimulation or (3) we might have overestimated the contraction rates. Spontaneous single contractions called ‘twitches’ are common during REM sleep. However, we only considered two or more successive contractions as responses, thereby eliminating all twitches. Moreover, behavioral responses were assessed blind to the sleep stage and to the stimulation period (ON versus OFF), ensuring that any putative false detection bias is uniformly distributed in all sleep stages and stimulation periods. Therefore, any differences in the response rates between ON and OFF periods reflect a genuine effect.

One might argue that the behavioral responses we observed during sleep occurred during brief episodes of wakefulness. Yet, all trials containing a micro-arousal (before and/or after the stimulation) were excluded from all analyses to ensure that participants were indeed asleep while responding, at least according to the well-accepted sleep scoring rules^29^. Moreover, EEG spectral measures in responsive sleep trials reflected the expected sleep stage variations, with significantly higher δ power and significantly lower α power compared to wake trials. Finally, poststimulus analyses revealed an increase in α and β power in responsive trials. However, given the frontal localization of these modifications and the timing relative to the motor preparation potentials, we argue that they reflect cognitive and motor processes rather than micro-arousals, at least in the classical sense. Recent studies suggest that the discrete frontiers between wake and sleep might be fuzzier than the international sleep criteria would allow^29^. For example, local sleep-like phenomena can be observed during wake and influence cognitive capacities and behavior^48,49^. In the same way, it is possible that our participants had ‘local wake events’ (in space and/or time) allowing them to respond to external stimuli while sleeping. Our current gold-standard sleep scoring guidelines are not suited to detect such subtle variations in brain dynamics. By calling into question the assumption that behavioral and coarse physiological measures of sleep always align, our study could precipitate the development of finer-grained sleep scoring that better captures cognitive capacities including behavioral responsiveness in the wake-sleep continuum. Such endeavor may be clinically relevant. For example, sleepwalking could be interpreted as extreme forms of these local wake events, still happening on a global background of sleep brain activity^50,51^.

While both participants with and without narcolepsy displayed responses during sleep, those with narcolepsy responded more. Because both groups had similar contraction rates during OFF periods, patients’ increased responsiveness is not due to an overall decrease in the detection criterion. Enhanced responsiveness in patients could be due to (1) an acquired capacity to remain connected with their surroundings while sleeping as an adaptation to their tendency to fall asleep in unconventional situations, (2) a reduced muscle atonia compared to healthy controls^52^ and (3) a higher proneness to experience ‘local wake events’ due to narcolepsy-related sleep-wake instability^52^. Even though participants with narcolepsy responded more frequently during sleep, both populations shared common EEG marker modifications in responsive trials. Crucially, the performance of a classifier trained with data from participants with narcolepsy generalized to HP. These two findings strongly suggest that the existence of these transient windows of behavioral reactivity is a general feature of sleep, of which narcoleptic participants present an exacerbated profile.

Our results enhance our understanding of the lucid dream phenomenon and of its neural correlates^47,53^. We found modifications in spectral power (increase in normalized PSD of γ and decrease in normalized PSD of δ) as well as an increase in signal complexity (SE) during lucid REM sleep, compared to nonlucid REM sleep, supporting the findings from a recent study^54^. Notably, we provide strong evidence that lucid participants perceived stimuli in a conscious manner. This evidence included subjective report (the gold standard for assessing conscious access) and the presence of neural responses previously shown to reflect conscious perception^43–45^ (a stable and sustained brain activity in response to stimuli). These results show that lucid dreaming is not only characterized by a reemergence of metacognitive and volitional capacities^55,56^ but also by a capacity to consciously process external information.

To what extent were nonlucid sleepers conscious when responding to stimuli remains an open question. Indeed, contrarily to lucid dreamers, nonlucid dreamers typically could not recall having performed the task during sleep and we could not perform temporal generalization decoding due to the insufficient number of trials in these participants. Either way, our findings have major consequences for consciousness research. If nonlucid sleepers unconsciously processed stimuli, the fact that they could make a lexical decision associated with a behavioral response would push further the boundaries of what is considered possible for an unconscious process. On the other hand, if nonlucid sleepers were actually conscious when responding, our experimental design could help in probing the minimal core of cortical activity required for conscious processing. In our opinion, several lines of evidence favor conscious processing in N2 and nonlucid REM sleep. First, neurophysiological markers computed before the stimulation in responsive trials were similar to the ones in lucid participants, suggesting that the neural state associated with responsiveness was comparable in both cases. Furthermore, the unconventionality of the response modality (corrugator or zygomatic muscle contractions) makes the automatization of the task difficult. Finally, reaction times to stimuli largely exceeded the one classically observed for automatic and unconscious processing (typically around 200 ms versus several seconds in our task)^57^. One may wonder why participants would fail to report having done the task if they had consciously performed it. We hypothesize that the rich neural states presumably allowing responsiveness need to be sustained over a certain time to be encoded. These rich states might have been less stable in nonlucid participants, as suggested by the difference in neurophysiological markers between responsive and nonresponsive trials (not found in lucid participants), preventing episodic memory encoding and thus subjective reports.

The standard view of sleep/wake states assumes that we would be either awake or asleep. Overall, our findings suggest that this view does not account for the richness and high variability within each of these states. This intuition goes along with a recent theory in the memory domain^58^, arguing that the high prevalence of aperiodic brain activity during sleep (up to 50% of brain activity without prominent oscillations, even in N3 sleep) could have a central role in processing internal stimuli (that is, imprinting memories into existing networks). Our results supplement this view by showing that access to external information might fluctuate even in traditionally defined states of consciousness (for example, a given sleep stage) depending on the ongoing brain activity. We could imagine sleep and wake as a continuum of stages whose physiology is more (for example, wake) or less (for example, N3 sleep) favorable for the emergence of the rich neural states that enable conscious access and behavioral response to external stimuli^59^.

Our study opens the way for many exciting studies investigating sleepers’ cognitive capacities and their associated phenomenology. By implementing a second probe about a participant’s current mental state, we could assess metacognition during responsive moments (for example, do sleepers know that stimuli come from the outside, or do they integrate them in their dream?). We could also test the extent to which the sleeping brain is able to acquire new information, hence fueling the debate on whether sleep learning is limited to conditioning^13^ and implicit memory processes^15^ or could extend to the formation of an explicit memory trace. By tracking how the neurophysiological markers indexing a rich cognitive state fluctuate in real time and by sending stimuli depending on their values, we could test the causal relationship between the neural state and responsiveness. Moreover, we could target these brief windows of reactivity in sleep to attempt real-time communication with individuals across different sleep stages, which would open the exciting possibility to inquire about any sleepers’ mental states beyond the particular case of lucid dream^19^. Our findings also raise questions relevant for clinical practice—are responsive periods during sleep less recuperative than unresponsive periods? Our methods could be used to investigate ‘sleep depth’ in patients suffering from excessive daytime sleepiness or for bringing mechanistic insights into the puzzling mismatch between subjective wake perception and classical sleep markers in paradoxical insomnia. By demonstrating the existence of windows of behavioral responsiveness across most sleep stages, our study provides a new tool for unlocking the mystery of what happens in sleepers’ minds.

## Methods

### Participants

#### Participants with narcolepsy

Thirty participants with narcolepsy were recruited for this study (14 women, mean age: 35 ± 11 years) from the patients followed in the National Reference Center for Narcolepsy in the Pitié-Salpêtrière Hospital. Twenty-four of them (80%) were frequent lucid dreamers who reported more than three lucid dreams per week on average (others reported less than one lucid dream per year). Participants met the international criteria for narcolepsy^60^, including (1) excessive daytime sleepiness occurring daily for at least 3 months; (2) a mean sleep latency lower than or equal to 8 min and two or more sleep onset REM sleep periods on the multiple sleep latency tests (five tests performed at 08:00, 10:00, 12:00, 14:00 and 16:00 and (3) no other better cause for these findings, including sleep apnea syndrome, insufficient sleep, delayed sleep phase disorder, depression and the effect of medication or substances or their withdrawal. They were required to pause their medication for the day of the experiment to facilitate sleep onset. We recruited patients with narcolepsy type 1 (*n* = 17, with clear cataplexy or hypocretin deficiency) and type 2 (*n* = 13, no cataplexy or hypocretin deficiency). Among the 30 participants, three (two women) were discarded from the analyses because of technical issues affecting the recordings. In total, data from 27 participants with narcolepsy (21 frequent lucid dreamers) were analyzed in this study.

#### HP

Twenty-two HP (all nonlucid dreamers) were recruited for this study (ten women, mean age: 24 ± 4 years). They had no or little experience with lucid dreaming (less than two lucid dreams in their lives). They had no sleep disorder and were in good shape, as assessed by a sleep clinician. To further facilitate sleep onset, we asked participants to sleep about 30% less than usual during the night preceding the experiment (either by going to bed later or waking up earlier) and to avoid stimulants on the day of the experiment. Fourteen went through the experiment in the morning and eight of them went through the experiment in the afternoon. One participant was discarded from the analysis because of technical issues affecting the recordings.

All participants were native French speakers and gave written consent to participate in the study. No statistical methods were used to predetermine sample sizes, but our sample sizes were similar to those reported in previous publications^8,21^. The protocol had been approved by the local ethics committee (CPP Ile-de-France 8). Participants with and without narcolepsy were paid €200 and €70, respectively, as compensation for their participation in the study (participants with narcolepsy also took part in an unrelated experiment the following day; the results of this second study are not described here).

### Experimental design

In this study, we tested participants’ ability to perceive, discriminate and respond to auditory stimuli while asleep. Participants lay in a bed in a sound-attenuated room in the sleep unit. They were asked to perform a lexical decision task in which words and pseudowords were verbally presented in a pseudorandomized fashion. Participants with narcolepsy went through five 20-min naps, with an 80-min break between each nap (Fig. 1). Before the experiment, participants underwent a short training (10 min) to familiarize themselves with the type of stimuli and the task (ten repetitions). Stimulus presentation volume was 48 dB on average and adjusted for each participant during the training period. Each nap session contained ten ‘ON’ stimulation periods during which six stimuli (three words and three pseudowords) were presented every 9–11 s on top of continuous white noise presented throughout the nap. Each stimulus was presented only once in the entire experiment. The ‘ON’ stimulation periods were separated by 1 min nonstimulation periods (OFF periods) during which only white noise was presented. Following a previously validated response paradigm during sleep^19^, participants were instructed to decide whether the stimulus was a word or a pseudo-word and indicate their response by making three, brief, successive contractions of either the corrugator (frowning) or the zygomatic (smiling) muscles, depending on the stimulus type (for example, contracting the corrugator if they heard a pseudo-word and the zygomatic if they heard a word). The muscle-stimulus association was counterbalanced across participants. Notably, the stimulation started when the participants were still awake, but participants were explicitly authorized to fall asleep while performing the task. They were asked to perform the task before falling asleep, if they woke up during a nap and if they heard the stimuli in their sleep. If participants were lucid dreaming but did not hear any stimuli (word or pseudowords), they were instructed to communicate their lucidity with a ‘mixed’ signal, alternating a single corrugator muscle and a single zygomatic muscle contraction. Note that we chose not to use the gold-standard method to signal lucidity here (left–right–left–right ocular code) for the following three reasons: (1) the ocular code ‘pollutes’ the EOG channel, which might lead to bias when scoring REM sleep, (2) several lucid dreamers with narcolepsy explicitly told us that facial codes were easier to perform, less disturbing of the ongoing dream, and less awakening than the ocular code and (3) our experiment required three different codes (one for each stimulus type and one for signaling lucidity if no sounds were heard). After each nap, participants were awakened by an alarm that rang until they pressed a button. They were asked to report ‘what was going through their mind’ before the alarm and indicate whether (1) they had a lucid dream, (2) they communicated their lucidity with the mixed signal, (3) they heard the stimuli during the nap, (4) they responded to the stimuli and (5) they remember any stimuli (word or pseudo-word) from the nap (free recall). Finally, participants performed an old–new recognition task, during which they were presented with stimuli they heard during the preceding nap and new stimuli that were never presented during the experiment. Participants had to indicate whether they had heard the stimuli during the preceding session with one of the following responses: (1) I heard it from the dream (for example, a person from their dream saying the word), (2) I heard it from the outside world (pronounced by the computer), (3) I am not sure I heard it, (4) I am sure I did not hear it. They responded by pressing the corresponding button without any time pressure. The four options were explained to the participants during training, before the first session.

HP went through the same procedure except that the five naps were combined into a single, longer, 100-min daytime nap.

### Stimuli

Stimuli were French words and pseudowords pronounced by a female voice, taken from the MEGALEX database^61^. All stimuli were controlled for their duration (690 ms), and the words were controlled for their frequency and valence. Five distinct lists (one for each nap session) of 60 stimuli (30 words and 30 pseudowords) were created for each participant in a randomized fashion. Participants heard each stimulus only once during the day. Stimuli were presented through speakers using the Psychtoolbox extension^62^ for MATLAB (MathWorks). Stimuli were played every 9–11 s (random uniform jitter) after a 60-s OFF period (without stimuli). Button-press responses in the old–new recognition task were collected through a regular keypad.

### Electrophysiological recording

EEG (ten channels: Fp1, Fp2, Cz, C3, C4, Pz, P3, P4, O1, O2, referenced to the right mastoid (A2 electrode); 10–20 montage), EOG (two channels, positioned above the right superior canthus and the left inferior canthus), EMG (one channel on chin muscle for sleep staging, one channel on zygomatic and one channel on corrugator muscles for recording participants’ behavioral responses) and electrocardiography (EKG, one channel) were continuously recorded during the nap sessions. All signals were recorded simultaneously at a 2,048 Hz sampling rate. EEG data were amplified through a Grael 4K PSG:EEG amplifier (Medical Data Technology, Compumedics).

### Sleep scoring and identification of muscular responses

#### Sleep scoring

Sleep stages were scored offline by a certified sleep expert according to established guidelines^29^ using Profusion software (Compumedics, Medical Data Technology). For scoring, the EEG and EOG signals were filtered between 0.3 and 15 Hz, the EMG and EKG signals were filtered between 10–100 Hz and 0.3–70 Hz respectively. A 50 Hz notch filter was applied on all channels. Sleep scoring was visually performed on 30-s time epochs, each scored as wakefulness, N1, N2, N3 or REM sleep, according to the American Academy of Sleep Medicine international rules. Detailed information on the sleep characteristics can be found in Supplementary Table 12. Micro-arousals were scored when α rhythm was present for more than 3 s and less than 15 s (if longer, the epoch was scored as wake) and, in REM sleep, when there was an increase in chin muscle tone in addition to the α rhythm. Trials containing micro-arousals were excluded from further analyses. A nap was considered lucid based on the subjective report (if the participant reported having a lucid dream during the nap). In this case, all REM sleep epochs of this nap were then considered as lucid REM sleep. Note that HP never reported having a lucid dream.

#### Identification of muscular responses

The recording of the nap was divided into 120 mini-epochs of 10 s. The sleep stage for each mini-epoch was defined by the sleep score of the corresponding 30-s epoch. Mini-epochs containing a micro-arousal were discarded from the analyses. The presence of zygomatic or corrugator muscle contractions was assessed visually, looking offline at the EMG signal for each mini-epoch. Notably, the scorer was blind to the sleep stage and to whether a stimulus was presented during the mini-epoch (corresponding to an ON period) or not (corresponding to an OFF period). Muscle contractions were considered as a response if they contained at least two consecutive contractions. Single contractions were considered as a twitch and scored as a no-response. To ensure the quality of the scoring, 10% of the data was later re-evaluated by another blind scorer who showed 84% consistency with the first scorer.

### EEG preprocessing and analysis

Only the EEG segments corresponding to the ‘ON periods’ were analyzed.

#### Preprocessing

Raw files were set to a right mastoid reference (A2 electrode).

Following the previous work^34^, raw EEG files were band-pass filtered between 0.1 and 45 Hz, with 50 and 100 Hz notch filters. Data were downsampled to 250 Hz. Trials were then segmented in the following way:

1. from −1,000 to 8,000 ms relative to stimulus onset for raw spectral analyses (PSDs in the prestimulus and poststimulus periods).
2. from −1,000 to 4,000 ms relative to stimulus onset for ERPs and time–frequency analyses.
3. from −350 to 1,700 ms for temporal generalization decoding against baseline analysis.
4. from −1,000 to 0 ms relative to stimulus onset for computation of electrophysiological markers of higher cognitive states and related machine learning analyses.

The obtained epochs were cleaned, based on their voltage maximum peak-to-peak amplitude, using a fully automatic procedure with the autoreject^63^ algorithm. The Python^64^ implementation of the autoreject algorithm allows for the automatic calculation of an optimal global rejection threshold for a set of epochs, using a cross-validated machine learning algorithm. For each wake/sleep stage in our data (wake, N1, N2, N3 and REM sleep), we calculated a separate global rejection threshold (the same for all participants in each group for a given sleep/wake stage) and we rejected all trials with at least one EEG channel exceeding the given threshold. Note that this drastic rejection method was associated with high rejection rates but ensured the quality of our data. More conservative automatic cleaning methods such as interpolation of bad channels were not applicable to our ten channels EEG montage. All epochs from two participants with narcolepsy were rejected due to our strict rejection criterion. Therefore, only 25 participants with narcolepsy were included in the EEG analyses.

All trials were labeled as belonging to a particular sleep/wake stage (wake, N1, N2, N3 or REM sleep) according to the sleep scoring described above (corresponding 10 s mini-epoch), as being responsive or nonresponsive according to the presence or absence of a valid behavioral response (corrugator or zygomatic muscle contraction) and as lucid or nonlucid according to the global label of the nap (cf. above).

### Automatized detection of spindles and slow waves

Raw EEG files were band-pass filtered between 0.1 and 45 Hz, with 50 and 100 Hz notch filters. Data were downsampled to 250 Hz. Trials were then segmented from −1,000 to 8,000 ms relative to stimulus onset. We analyzed N2 trials of HP using a previously validated automatized sleep scoring algorithm (YASA^64^). For each trial (from −1,000 to 8,000 ms relative to stimulus onset) we assessed whether at least one spindle or slow wave (independently for each one of these two sleep graphoelements) was present during the duration of the trial, in at least two different channels. We then computed for each sleep graphoelement, each participant and each condition (responsive versus nonresponsive trials), the proportion of trials containing graphoelements (spindles or slow waves). We studied the effect of responsiveness (responsive versus nonresponsive) and sleep/wake stage (N2 sleep versus wake), as well as their interaction, over the proportion of trials containing at least one spindle or slow wave (independently for these two sleep graphoelements), using repeated measures analysis of variance. We corrected *P* values for lack of sphericity using a Greenhouse–Geisser correction.

### Spectral analyses of prestimulus and poststimulus periods

We computed the PSDs in δ (1–4 Hz) and α (8–12 Hz) frequency bands, using Welch’s method. The length of each Welch segment (windowed with a Hamming window) was set to be equal to the length of the fast Fourier transform and equal to 250 samples (1,000 ms). The obtained segments were then averaged, to obtain a single value per epoch, channel and frequency. To obtain the normalized PSDs in each frequency band of interest (α and δ), we (1) added the raw power of all the frequencies in each frequency band of interest; (2) computed for each trial and each electrode the normalized PSD by normalizing the raw frequency band PSD by the total power of the given electrode and (3) averaged all the channels to obtain a single PSD value per frequency band per trial.

### Time–frequency analysis

We computed single-trial stimulus-locked time–frequency representation for each group and each sleep/wake stage using Morlet wavelets. We choose the wavelets frequencies (*n* = 30) on a logarithmic scale with a lower bound of 2 Hz and an upper bound of 30 Hz (frequencies: 2, 2.2, 2.4, 2.6, 2.9, 3.2, 3.5, 3.8, 4.2, 4.6, 5, 5.6, 6.1, 6.7,7.4,8.1, 8.9, 9.8, 10.7, 11.8, 12.9, 14.2, 15.6, 17.1, 18.8, 20.6, 22.7, 24.9, 27.3 and 30 Hz). The number of cycles was adapted to each frequency (n_cycles = frequency/2). For computational reasons, we applied a decimation factor of 2 before conducting this analysis. We obtained a time–frequency power matrix for each trial and each electrode. We then applied a log-ratio baseline correction relative to the −1,000 to 0 ms time period. For statistical analysis on predefined frequency bands (δ (2–4 Hz), α (8–12 Hz) and β (12–30 Hz)), we extracted the total power in the given frequency band for each time sample and conducted a mass-univariate analysis over the time dimension for each electrode (Statistical analysis).

For response-locked time–frequency analysis, we realigned the baseline-corrected time–frequency matrices relative to the behavioral response onset. The new realigned trials spanned from −1,500 to 1,000 ms relative to response onset (we dropped for further analysis all trials with insufficient time points either before or after response onset).

### Response-locked ERPs

After baseline correction (−1,000 to 0 ms relative to stimulus onset), we realigned time-domain signals of responsive trials relative to the behavioral response onset. We then averaged trials to obtain ERPs, independently for each group and each sleep/wake stage. For visualization purposes, we applied a low-pass filter of 10 Hz before plotting the obtained response-locked ERPs.

### Stimulus-locked ERPs

For each group (HP and NP) and for each sleep/wake stage, we averaged stimulus-locked trials to obtain ERPs (after baseline correction relative to the −1,000 to 0 ms time period). We then conducted a mass-univariate analysis on time dimension, independently for each EEG channel, using mixed-linear models with responsiveness as the explanatory factor, and participant ID as a random effect. We corrected *P* values for multiple comparisons using an FDR procedure. Results are presented in Supplementary Fig. 14. For visualization purposes, we applied a low-pass filter of 10 Hz.

### Calculation of electroencephalographic markers tracking cognitive modifications

Previous work has shown that cognitive and consciousness state modifications can be tracked using different spectral, connectivity or complexity measures derived from the scalp or intracranial electroencephalographic recordings. By combining these markers, it is possible to distinguish conscious participants, patients in a minimal consciousness state and patients with unresponsive wakefulness syndrome^34,36^. These measures can also differentiate sleep stages (REM sleep and wakefulness versus N3)^38^ and track cognitive and consciousness modifications related to psychedelics or meditation^35^.

In our study, we selected the following three types of measures among those markers:

1. Spectral measures—we computed the normalized PSDs in δ (1–4 Hz), θ (4–8 Hz), α (8–12 Hz), β (12–30 Hz) and γ (30–45) frequency bands using the same methods described above.
2. Connectivity measures—we computed the wSMI, a functional connectivity measure capturing linear and nonlinear coupling between sensors, which relies on the symbolic transformation of the EEG signal. We computed the wSMI in the θ band (4–8 Hz)^36^. The choice of the θ frequency band was based on previously reported results^34,36^, showing that the wSMI calculated on this frequency band was the most efficient in detecting residual consciousness in brain-injured patients with a disorder of consciousness.
3. Complexity measures—we computed three different complexity measures, the KC, the PE *θ* and the SE.

See Supplementary Material in ref. ^34^ for a detailed description of each measure and its computation. Details regarding the SE can be found in ref. ^65^.

Each one of the previously described markers was computed during the 1,000 ms time window preceding the presentation of the stimulus (word or pseudo-word), during the ON periods, independently for each participant, trial and for every electrode (*n* = 10) or pair of electrodes (*n* = 45) for the wSMI. A wSMI global score for each electrode was computed by calculating the median connectivity of each electrode with all the other electrodes. Finally, for each participant and each trial, each marker was summarized by calculating the mean across channels, resulting in a single scalar per marker per trial.

### Prediction of responsiveness using a decision tree algorithm

We aimed at predicting, independently for each sleep/wake stage, if a given trial would contain a response or not based on the EEG markers computed during the 1,000 ms time period preceding the stimulus presentation. We used a random forest algorithm, a classification algorithm consisting of many decision trees. This algorithm implements bootstrapping and feature randomness when building each tree, which ensures the construction of an uncorrelated forest of trees. Because the different trees in the forest are uncorrelated, their global prediction by committee is more accurate than that of any individual tree. Random forest has shown to be among the best currently used machine learning classifiers, in a very wide range of different datasets (*n* = 112) from several research fields^66^, outperforming other choices as SVM classifiers.

We conducted an independent analysis for each sleep/wake stage. For each trial, the classifier was provided with ten features, as well as the label (‘responsive’ versus ‘nonresponsive’) of the trial. The ten features were the nine EEG markers described in the previous section and the participant identity. The random forest classifier was composed of 100 estimators (trees). Because our data were unbalanced in terms of the number of responsive trials compared to nonresponsive ones, the weights of each class were adjusted in an inversely proportional manner to class frequencies.

The following two different training/testing strategies were used:

- For the participants with narcolepsy, we used for each stage a standard tenfold stratified cross-validation procedure. In each fold, data were split into training (9/10 of the trials) and testing (1/10 of the trials) sets, in a manner that preserved class frequencies in each split. Trials of each class were shuffled before splitting in a pseudorandomized manner. In each fold, the predictions of the classifier for the testing set were used to compute the balanced accuracy score and the *F*1 score of the classifier (see definition and method for calculation of these scores below). We then computed the mean balanced accuracy and F1 scores across folds, as well as their confidence interval. F1 scores can be found in Supplementary Fig. 15 and Supplementary Table 9.
- For the participants without narcolepsy, because responsive trials were scarce in particular during N2 sleep and REM sleep, we decided to train our classifier with the data of the participants with narcolepsy and to test its performance on data from participants without narcolepsy. Specifically, we fitted our classifier with the N2 sleep trials from participants with narcolepsy and then tested its predictions on N2 sleep trials from the participants without narcolepsy. As before, we computed balanced accuracy and F1 scores. To obtain a distribution of scores in the absence of cross-validation, we repeated the fitting and testing steps ten times (note that the random parameters of the random forest classifier allowed us to obtain a distribution of—closely related—scores in this manner).

As mentioned above, we computed two scores to measure the performance of our classifier, both measures being well adapted to unbalanced datasets^67^ as ours (with more nonresponsive trials than responsive ones during sleep):

- The balanced accuracy score corresponds, in binary classification problems, to the mean of the sensitivity—also called recall (‘How many relevant items are retrieved?’) and the specificity (‘How many nonrelevant items are correctly identified’). In terms of true positives (TP), false negatives, true negatives (TN) and false positives (where, in our case, TP are responsive trials correctly identified by the classifier, and TN nonresponsive trials correctly identified by the classifier), the balanced accuracy score can be computed by the following formula:
  $${\mathrm{Balanced}}\; {\mathrm{accuracy}}=\frac{\frac{{\mathrm{TP}}}{{\mathrm{TP}}+{\mathrm{FN}}}+\frac{{\mathrm{TN}}}{{\mathrm{TN}}+{\mathrm{FP}}}}{2}$$
- The *F*1 score corresponds, in binary classification problems, to the harmonic mean of the precision (‘How many retrieved items are relevant?’) and the sensitivity. It can be computed by the following formula:

$$F1\,{\mathrm{score}}=\frac{2}{{\left(\frac{{\mathrm{TP}}}{{\mathrm{TP}}+{\mathrm{FN}}}\right)}^{-1}+{\left(\frac{{\mathrm{TP}}}{{\mathrm{TP}}+{\mathrm{FP}}}\right)}^{-1}}$$

We first run an analysis taking into account all responses (correct and incorrect). Then, we separately studied the prediction accuracy when considering only correct or incorrect responses.

### Decoding of stimulus-related brain activity

We aimed at assessing brain responses to stimuli in function of participants’ sleep/wake stages and of their responsiveness to the task using a multivariate pattern analysis with the temporal generalization decoding method^43^. The idea of this analysis is to test, for a given time point after stimulus presentation, how different the multivariate pattern of activity across electrodes was at this specific time point compared to the pattern at baseline (before stimulus presentation), for the different conditions.

To reduce computation time, we first downsampled our data to 100 Hz (decimation factor of 2.5). To ensure a correct features/number of trials ratio, we restricted our analysis to three centroparietal electrodes (Cz, Pz and P3), and, for each condition (sleep stage/responsiveness), we only included in our analysis the participants who had at least 15 trials of the given condition. Given these restrictions, we only had enough participants for statistical analysis for lucid REM sleep (ten participants for responsive trials and nine participants for nonresponsive trials) and for wake (14 participants for responsive trials). Then, for each condition, participant, trial and channel, we computed the mean voltage during the 350 ms baseline period before stimulus presentation and used this value to create dummy ‘baseline’ trials with the same dimensionality as the original trials. Note that after this step, for each condition and each participant, we obtained a balanced set of dummy ‘baseline trials’ (reflecting baseline brain activity before/without stimulus presentation) and actual trials where the stimulus was presented.

Then, independently for each condition and each participant, we trained a linear classifier to decode stimulus-present versus stimulus-absent trials (‘baseline’ dummy trials versus actual trials), using an L2-regularized (*C* = 1) logistic regression, in a fivefold cross-validation procedure. In each fold, all the trials were shuffled in a pseudorandomized manner and split into a training set (4/5 of the trials) and a testing set (1/5 of the trials). The features (channel amplitudes) were standardized across training trials before being provided to the classifier for training. This training procedure was applied at each time step independently. Following the time-generalization approach, the model trained at each time step was then tested at all the time steps on the testing set trials, at each cross-validation fold. The classifier performance at each training and testing time was evaluated by the area under the receiver operating curve (AUC) at each cross-validation fold. At the end of the cross-validation procedure, the global performance of the classifier at each training and testing time was obtained by averaging the intermediate values obtained at each fold, for each participant and each experimental condition. Group-level performance for each condition was finally obtained by averaging across participants, independently for each condition (stage/responsiveness).

### Statistical analysis

Most statistical analyses were conducted in R^68^ using the lme4 (ref. ^69^), emmeans^70^, BayesFactor^71^, car^72^ and DHARMa^73^ packages. For the machine learning analysis, statistics were conducted in Python^64^ using the numpy^74^, scipy^75^, pingouin^76^ and scikit-learn^77^ packages. All statistics were corrected for multiple comparisons using the FDR Benjamini–Hochberg procedure. FDR corrections were applied separately to each group of statistical tests (each panel in the Figs. 3–6, and 8). For example, one correction was performed for Fig. 3a, combining NP and HP and all sleep stages. Assumptions of the generalized linear models were evaluated using DHARMa^73^ package in R^68^. For linear mixed models, the distributions of residuals as well as Q–Q plots were visually inspected but were not formally tested. The significance of single factors was tested with Wald *χ*^*2*^ tests using car^72^ package. When the statistics were computed at the participant level rather than at the single-trial level, the observations were weighted according to the number of trials that each participant had.

#### Behavior

Linear mixed models with participant ID as a random factor (random intercept) were used for all statistical analyses. We evaluated participants’ ability to respond to stimuli in different sleep stages (Figs. 1 and 3). First, we focused on the comparison between the ON and OFF periods separately for each sleep stage. Binomial generalized linear mixed models with stimulation period (ON versus OFF) as the independent variable and responsiveness (response versus no response; both contraction types combined) as the dependent variable were used in this analysis. Next, we focused on the ON stimulation periods during which participants were presented with stimuli. The model had sleep stages (wake, N1, N2, N3, REM sleep in HP and wake, N1 N2, N3, nonlucid REM and lucid REM sleep in participants with narcolepsy) as the independent variable and responsiveness (response versus no response) as the dependent variable. For accuracy, we computed the percentage of correct responses for each participant at each sleep stage and compared them to the 50% chance level using the Wilcoxon signed-rank test. Only participants with at least three responses were included in this analysis. Finally, the differences in reaction times in different sleep stages were assessed using a linear mixed model (Fig. 4). An inverse transformation was applied to the reaction times (1/RT) to better fit the model assumptions.

#### Bayesian statistics

Bayes factors were computed using default settings of BayesFactor^71^ package as implemented in Python^64^. For detailed information about priors and settings, refer to the documentation.

#### EEG markers (spectral, connectivity and complexity)

To investigate how different neural markers differ in trials with a response and without any response, we first *z*-scored marker values at the participant level. We then used a mixed-linear model for each EEG marker with participant ID as a random factor (random intercept), responsiveness as the independent variable and the EEG marker as the dependent variable. The analysis was conducted at a single-trial level. Because responsiveness and sleep stages were not independent (for example, in wake we observed more responses than in N2 sleep), we could not include sleep stage as an additional independent variable in the models. Thus, we performed the tests separately for each sleep stage, resulting in a test for each marker in each sleep stage. We performed a similar analysis to compare, in REM sleep, lucid and nonlucid trials.

#### Time–frequency analysis

We conducted a mass-univariate analysis over the time dimension on preselected frequency bands of interest (δ, α and β), using mixed-linear models with responsiveness as the independent explanatory factor and participant ID as a random factor (the power in each time sample being the dependent variable). This analysis was conducted independently for each group, sleep/wake stage and electrode. A correction for multiple comparisons was applied using the FDR procedure.

#### Prediction of responsiveness at a trial level using a random forest classifier

We scored classifier performance at each sleep/wake stage and for each group using the balanced accuracy score and the *F*1 score (cf above). To assess how different these scores were from chance level, we performed, independently for each score, a 500-permutation procedure. At each permutation, trial labels (responsive versus nonresponsive) were randomly shuffled, and the entire tenfold cross-validation procedure was performed, allowing us to obtain a distribution of chance-level scores. To calculate the *P* value for each state, we counted the number of permutation scores equal or higher to our true score and divided it by the number of permutations plus one.

#### Decoding of stimulus-related brain activity using temporal generalization decoding

For each experimental condition (sleep stage/responsiveness), classification performance at each training and testing time was tested against 0.5 (chance) using a two-sided nonparametric sign test across participants, and these statistics were then corrected for multiple comparisons using the FDR Benjamini–Hochberg procedure. In Fig. 8b, significant time points (*P* < 0.05 FDR corrected) with an AUC > 0.5 are outlined in black.

### Reporting summary

Further information on research design is available in the Nature Portfolio Reporting Summary linked to this article.

## Online content

Any methods, additional references, Nature Portfolio reporting summaries, source data, extended data, supplementary information, acknowledgements, peer review information; details of author contributions and competing interests; and statements of data and code availability are available at 10.1038/s41593-023-01449-7.

### Supplementary information

Supplementary Information Supplementary Note, Supplementary Results, Supplementary Figs. 1–15 and Supplementary Tables 1–12.

Reporting Summary

### Source data

Source Data Fig. 3 Raw behavioral data for both HP and narcoleptic participants (NP). Each row corresponds to a trial, each column gives information about the corresponding trial (for example, participant ID, sleep stage, response, etc.).

Source Data Fig. 4 Raw behavioral data for both HP and narcoleptic participants (NP). Each row corresponds to a trial, each column gives information about the corresponding trial (for example, participant ID, sleep stage, response, etc.).

Source Data Fig. 5 EEG marker values (spectral, complexity, etc.) at the trial level computed both at the prestimulus and poststimulus time periods. For each trial, the CSV files also indicate the participant’s ID, group (healthy or narcoleptic), sleep stage and response.

Source Data Fig. 6 EEG marker values (spectral, complexity, etc.) at the trial level computed during the −1 to 0 s time window (relative to stimulus presentation). For each trial, the CSV file also indicates the participant’s ID, group (healthy or narcoleptic), sleep stage and response. The second contains the results of the statistical analysis (contrast between responsive and nonresponsive trials, linear mixed model) for each group of participants, each sleep stage and each EEG marker.

Source Data Fig. 7 The balanced accuracy scores of the random forest classifier (trained to classify trials based on the presence of response) for each fold (*n* = 10) and each sleep stage, when trained with only correct responses or only incorrect responses.

Source Data Fig. 8 EEG marker values (spectral, complexity, etc.) at trial level computed during the −1 to 0 s time window (relative to stimulus presentation). For each trial, the CSV file also indicates the participant’s ID, group (healthy or narcoleptic), sleep stage and response.

Source Data Extended Data Fig. 4 Results, for each threshold and each window size, of the automatized response detection algorithm. For each trial, the predictions of the algorithm are presented for each possible combination of parameters. Window size is expressed in a number of samples (sampling rate 250 Hz).

Source Data Extended Data Fig. 5 Raw behavioral data for both HP and narcoleptic participants. Each row corresponds to a trial, each column gives information about the corresponding trial (for example, participant ID, sleep stage, response, etc.).

Source Data Extended Data Fig. 7 Statistical analysis (mixed-linear model, contrast between responsive and nonresponsive trials) for each frequency band (δ, α, σ and β), for participants with narcolepsy and HP.

Source Data Extended Data Fig. 9 EEG marker values (spectral, complexity, etc.) at the trial level computed during the −1 to 0 s time window (relative to stimulus presentation). For each trial, the CSV file also indicates the participant’s ID, group (healthy or narcoleptic), sleep stage and response.

Source Data Extended Data Fig. 10 EEG marker values (spectral, complexity, etc.) at trial level computed during the −1 to 0 s time window (relative to stimulus presentation). For each trial, the CSV file also indicates the participant’s ID, group (healthy or narcoleptic), sleep stage and response.

## Data Availability

All data that support the findings of the study can be found in OSF (https://osf.io/gbtjd/?view_only=254e0addb97a4b108e2fe35cce076799). Source data are provided with this paper.

## Code availability

Examples of custom analysis scripts can be found in OSF (https://osf.io/gbtjd/?view_only=254e0addb97a4b108e2fe35cce076799*)*.
