# Supplementary material for: Behavioral and brain responses to verbal stimuli reveal transient periods of cognitive integration of the external world during sleep
Source: Nat Neurosci. 2023 Oct 12;26(11):1981–93. doi: 10.1038/s41593-023-01449-7 (PMC10620087; doi:10.1038/s41593-023-01449-7)
Supplement: Supplementary file 1 — Supplementary Note, Supplementary Results, Supplementary Figs. 1–15 and Supplementary Tables 1–12. [file 41593_2023_1449_MOESM1_ESM.pdf]

nature neuroscience

Article

https://doi.org/10.1038/s41593-023-01449-7

Behavioral and brain responses to verbal stimuli reveal transient periods of cognitive integration of the external world during sleep

In the format provided by the authors and unedited

SUPPLEMENTARY INFORMATION

SUPPLEMENTARY TEXT

Automatic response detection algorithm

In addition to our visual evaluation of response contractions, we also detected behavioral responses using an objective, automatized method. To do so, we created a response detection algorithm based on voltage variance in our two EMG channels of interest (corrugator and zygomatic muscles). This algorithm computed the signal variance in the 1 second pre-stimulus baseline period and compared it to the post-stimulus variance using sliding-windows. Our algorithm had two predefined parameters: (1) the length of the sliding window and (2) a constant *k.* If the variance in a given sliding window exceeded k*baseline variance, the algorithm labeled the trial as responsive; otherwise, the trial was labeled as unresponsive. We ran the algorithm with different combinations of parameters. We used 1 and 2 second time windows (parameter 1), based on the observation that a unique muscle contraction took approximately 300ms. The second parameter k was either 5, 7, or 10. Examples of muscle contractions and corresponding variance modulations are shown in Extended Data Fig. 3.

We performed two analyses based on the response scoring of the automatic algorithm. First, we compared the concordance of our visual scoring to the one of the algorithm in different sleep stages using different performance metrics (accuracy, recall, and precision). For this analysis, we considered our visual scoring the gold-standard. Recall (or sensitivity) corresponds, in the context of our task, to the number of correctly labeled responsive trials divided by the total number of responsive trials (True Positives/[True Positives + False Negatives]). Here, true positives correspond to trials when both the automatic and visual scoring agree that there was a response; false negatives correspond to trials when the automatic scoring said there was no response whereas our visual scoring said there was one. Precision, on the other hand, corresponds to the number of correctly labeled responsive trials divided by the total number of trials labeled as responsive (True Positives/[True Positives + False Positives]). Here, false positives correspond to trials when the automatic scoring said there was a response whereas our visual scoring said there was none. We then tested the significance of these metrics against chance-level using a 500-permutations procedure (i.e. shuffling the 'responsive' and 'non-responsive' labels of our visual scoring 500 times, independently for each sleep/wake stage). We found, for all metrics and all tested sleep stages, a significant match between the two scoring methods, with accuracy ranging from 0.75 to 0.91. The detail of the results can be found in Supplementary Table S2 (for healthy participants) and in Supplementary Table S3 (for participants with narcolepsy). We then compared the response rate, as scored by the algorithm (blind to the visual scoring), in ON versus OFF stimulation periods. We found significant more responses during ON vs. OFF periods in all sleep stages in both populations (except in N3 for healthy subjects), replicating the results from our manual scoring. The details of the results based on different parameter combinations can be found in Extended Data Fig. 4. Since the two scoring methods were largely congruent and because we do not have a gold-standard to validate the objective performance of our algorithm (besides our visual scoring), we chose to keep our visual scoring for the rest of the analyses.

SUPPLEMENTARY RESULTS

Increased responsiveness is associated with increased accuracy

Since a negative correlation between accuracy and response rate could be a sign of false detection, we tested the relationship between these two behavioral measures. The relationship between the response rate and accuracy was evaluated at the participant level for each sleep stage and group using Pearson's correlation. Only participants with at least three trials were included in this analysis. Our analyses revealed that response rate was positively correlated with accuracy in participants with narcolepsy during Wake (R = 0.4, p = 0.04), N2 sleep (R = 0.49, p = 0.015), REM sleep (R = 0.6, p = 0.038) and lucid REM sleep (R = 0.64, p = 0.001). We also observed a similar tendency in healthy participants during Wake (R = 0.38, p = 0.094) and N1 sleep (R = 0.46, p = 0.062).

Old/New recognition task upon awakening

After each nap, participants performed an old-new recognition task, during which they were presented with stimuli they heard during the preceding nap and new stimuli that were never presented during the experiment. Participants had to indicate whether they had heard the stimuli during the preceding session with one of the following responses: 1: I heard it from the dream (for example, a person from their dream saying the word), 2: I heard it from outside (pronounced by the computer), 3: I am not sure I heard it, 4: I am sure I did not hear it. They responded by pressing the corresponding button without any time pressure. The four options were explained to the participants during training, prior to the first session. We assessed whether participants were able to correctly recognize the stimuli upon awakening. We focused on participants with narcolepsy (NP) since they went through 5 short naps, which should make the recognition of stimuli easier than in healthy participants who had a longer, 100 min nap. First, we computed, for each nap, the percentage of false recognition of new stimuli. A stimulus was considered "recognized" if participants reported either (1) hearing it in their dreams or (2) hearing it from outside of their dreams while asleep. On average 8.46% of new stimuli were falsely recognized. Then, we assessed the correct recognition of stimuli that were previously presented in different sleep stages. The percentage of correct recognition was 21.9% in Wake, 15.71% in N1, 8.6% in N2, 9.3% in REM, and 9.43% in lucid REM sleep. This percentage of correct recognition was significantly different than false recognition only in Wake ($p < 0.0001$, $z = 4.16$) and N1 sleep ($p < 0.002$, $z = 3.7$). Low recognition rates (even in Wake and lucid REM sleep trials) can be due to several factors. First, participants had no explicit instruction to remember the stimuli; they were simply asked to perform a lexical decision task. Moreover, each nap included a high number of stimuli (60 in each nap for the NP and 300 in the nap for the HP). Recalling such a high number of stimuli would already be an intense challenge for fully awake participants who would actively try to encode the stimuli. All stimuli were only played once throughout the experiment (no repetition of the same stimulus) and half of the stimuli were pseudo-words (harder to encode than words). And finally, the memory test was performed at the end of a nap, so in most cases long after the stimuli were played. For all these reasons, we believe that the explicit recognition task was far too difficult. It is possible that an implicit test would have been more suited to detect evidence of learning during sleep (Züst et al., 2019) than an explicit one.

Classical sleep graphoelements

We quantified the occurrence of classical sleep graphoelements (spindles and slow-waves) in non-responsive and responsive NREM sleep trials. Given that a manual quantification could be considered redundant with the polysomnographic scoring, we decided to use a previously validated automatized detection algorithm[30]. Since this algorithm has not been validated in patients with narcolepsy, we only analyzed data from HP, and more precisely N2 sleep trials (responsive and non-responsive), as well as Wake trials as a comparison. For each condition and each participant, we computed the proportion of trials including at least one spindle or one slow wave (independently for these two NREM sleep hallmarks), in the -1000 to 8000 ms time-window relative to stimulus-onset. While both spindle and slow wave occurrence (as detected by the algorithm) were modulated by sleep stage (Spindles: Wake 15% (+/-21%) *vs.* N2 37% (+/-30%), $F(1,12) = 5.7$, $p = 0.03$; Slow-waves: Wake 0.02% (+/-0.05%) *vs.* N2 22.4% (+/-21%), $F(1,12) = 14.3$, $p = 0.003$), we did not find a significant main effect of responsiveness (Spindles: responsive 25% (+/-31%) *vs.* non-responsive 26.8 % (+/-24.5%), $F(1,12) = 0.47$, $p = 0.5$; Slow waves: responsive 0.07% (+/- 0.18%) *vs.* non-responsive 0.17% (+/-0.17%), $F(1,12) = 2.7$, $p = 0.13$) nor an interaction with the sleep stage (Spindles: $F(1,12) = 0.06$, $p = 0.8$; Slow-waves: $F(1,12) = 1.5$, $p = 0.24$). In sum, we did not find evidence for a reduced occurrence of classical sleep graphoelements in responsive sleep trials, compared to non-responsive ones in HP.

SUPPLEMENTARY FIGURES

Supplementary Figure S1 Example of responses from a healthy participant during N2 sleep. All raw EEG and behavioral data are available on OSF (see Data Availability statement).

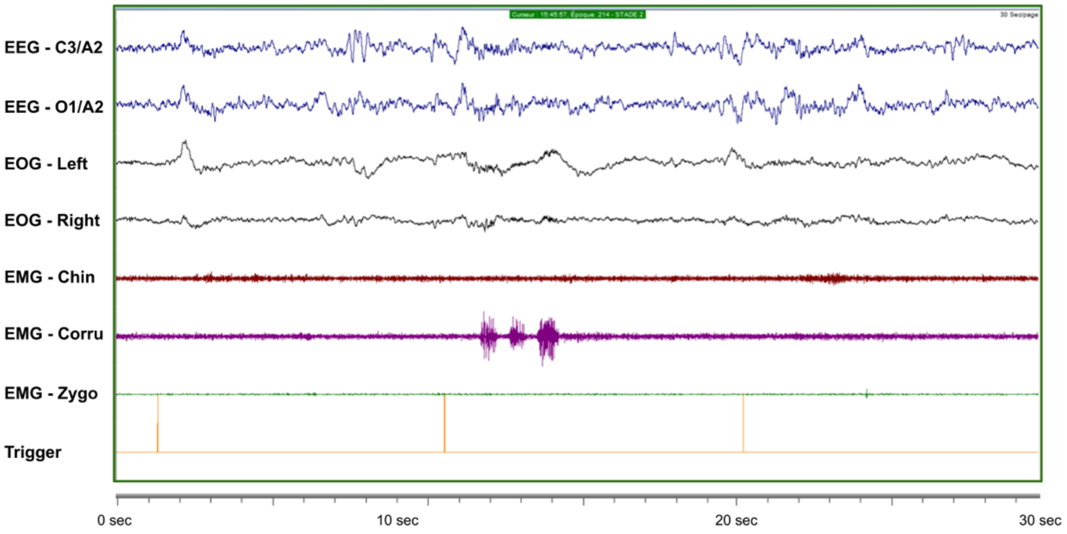

Supplementary Figure S2 Example of responses from a healthy participant during N2 sleep. All raw EEG and behavioral data are available on OSF (see Data Availability statement).

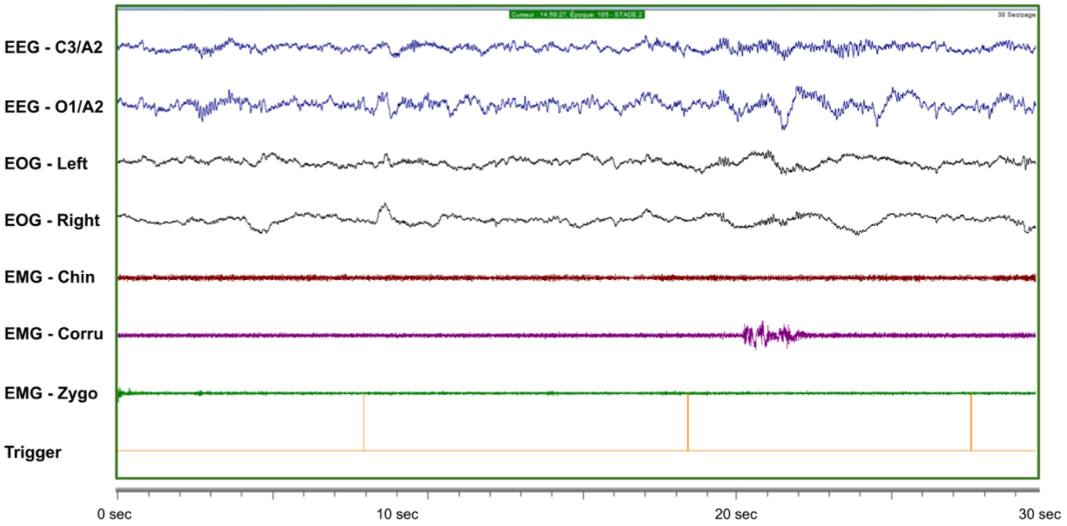

Supplementary Figure S3 Example of responses from a healthy participant during N2 sleep. All raw EEG and behavioral data are available on OSF (see Data Availability statement).

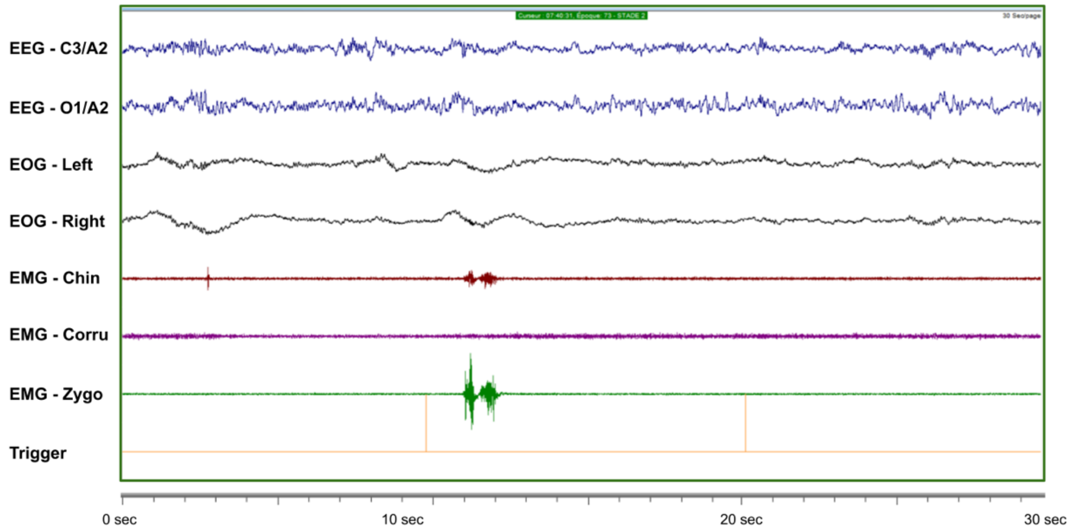

Supplementary Figure S4 Example of responses from a healthy participant during N2 sleep. All raw EEG and behavioral data are available on OSF (see Data Availability statement).

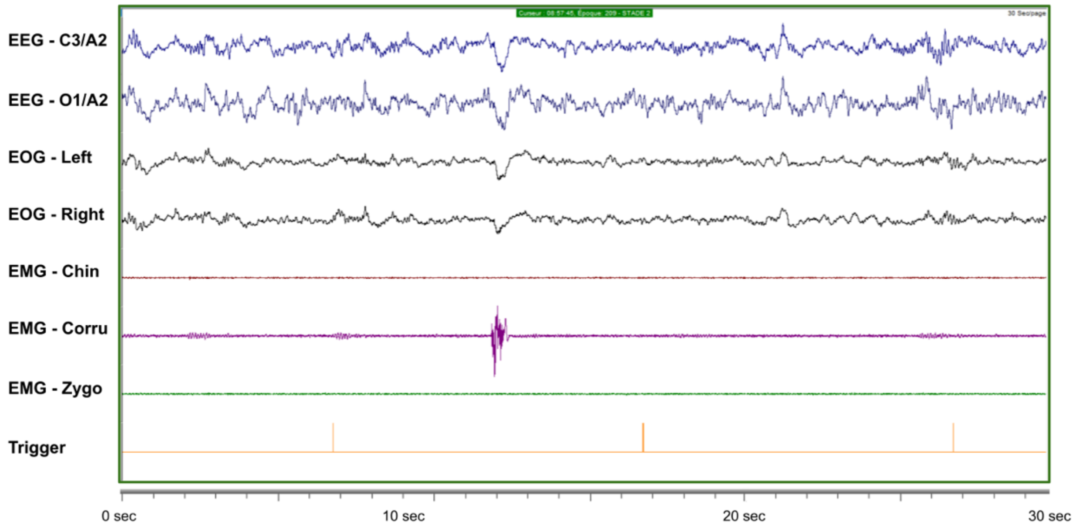

Supplementary Figure S5 Example of responses from a healthy participant during N2 sleep. All raw EEG and behavioral data are available on OSF (see Data Availability statement).

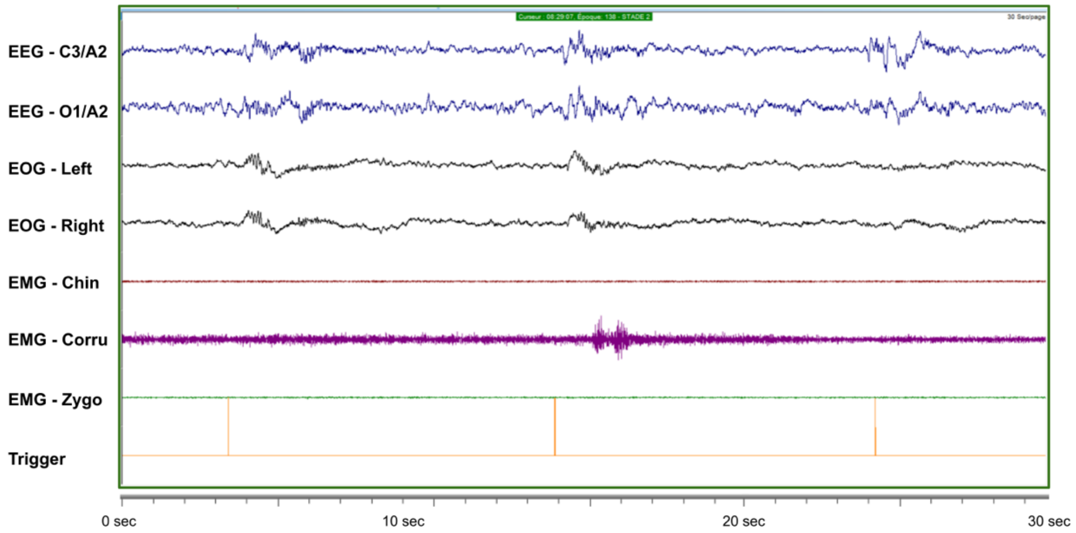

Supplementary Figure S6 Example of responses from a healthy participant during REM sleep. All raw EEG and behavioral data are available on OSF (see Data Availability statement).

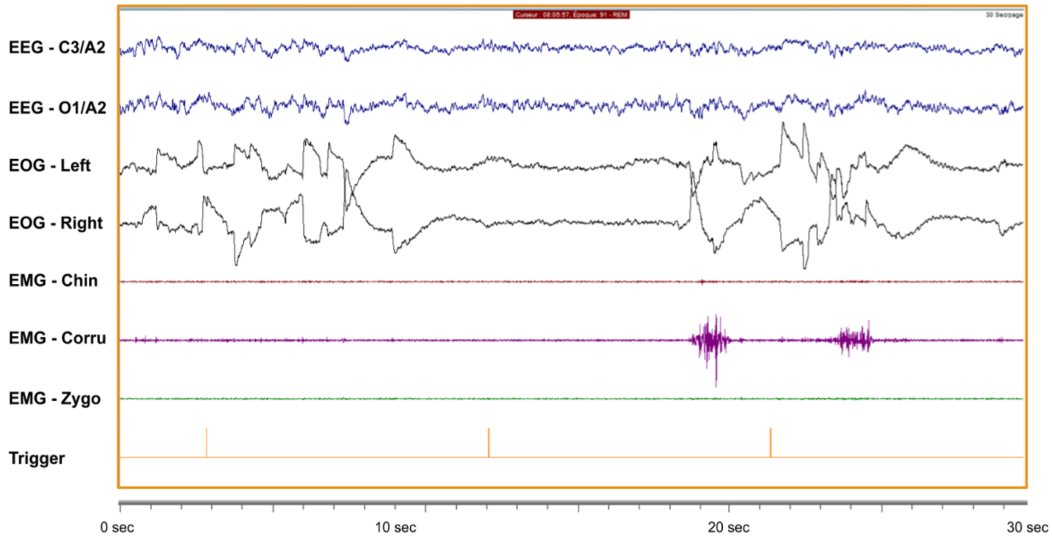

Supplementary Figure S7 Example of responses from a healthy participant during REM sleep.. All raw EEG and behavioral data are available on OSF (see Data Availability statement).

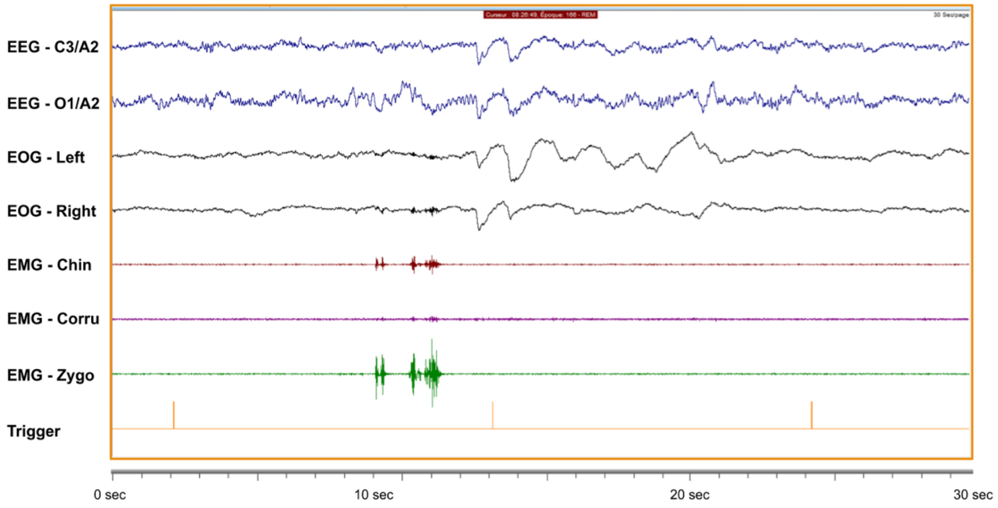

Supplementary Figure S8 Example of responses from a healthy participant during REM sleep.. All raw EEG and behavioral data are available on OSF (see Data Availability statement).

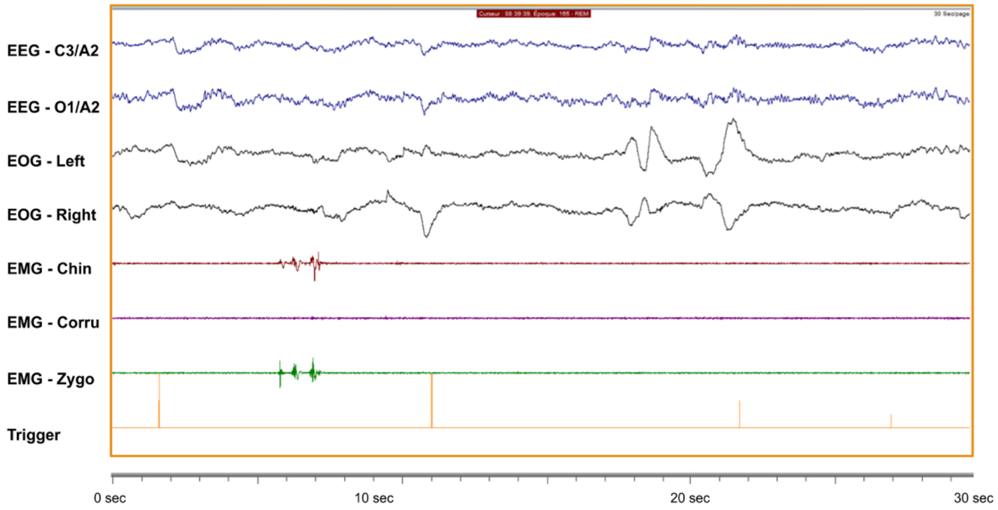

Supplementary Figure S9 Example of responses from a healthy participant during REM sleep.. All raw EEG and behavioral data are available on OSF (see Data Availability statement).

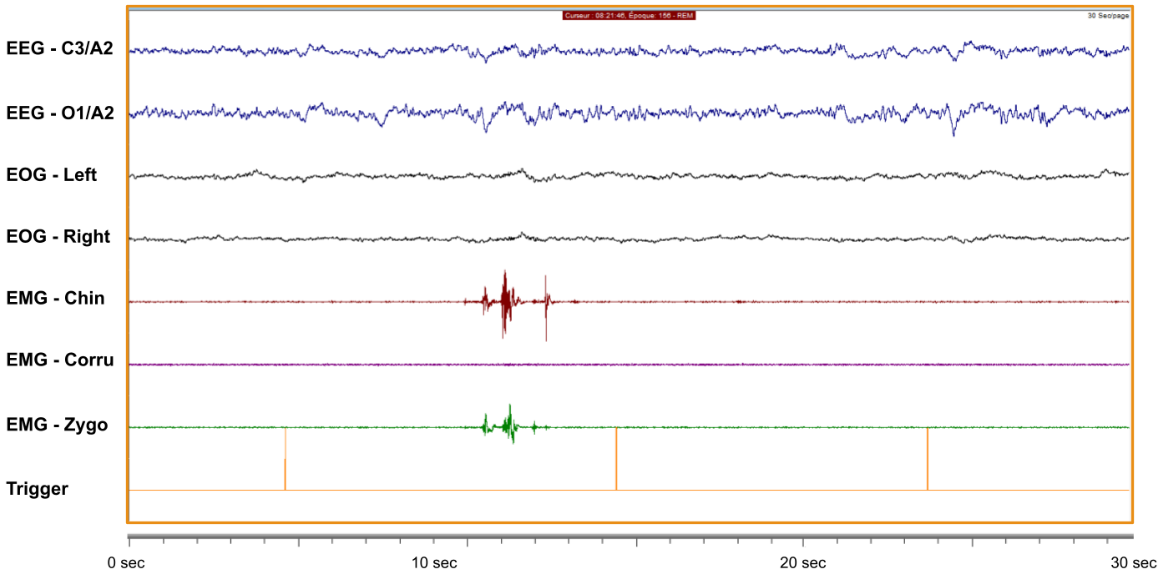

Supplementary Figure S10 Example of responses from a healthy participant during REM sleep.. All raw EEG and behavioral data are available on OSF (see Data Availability statement).

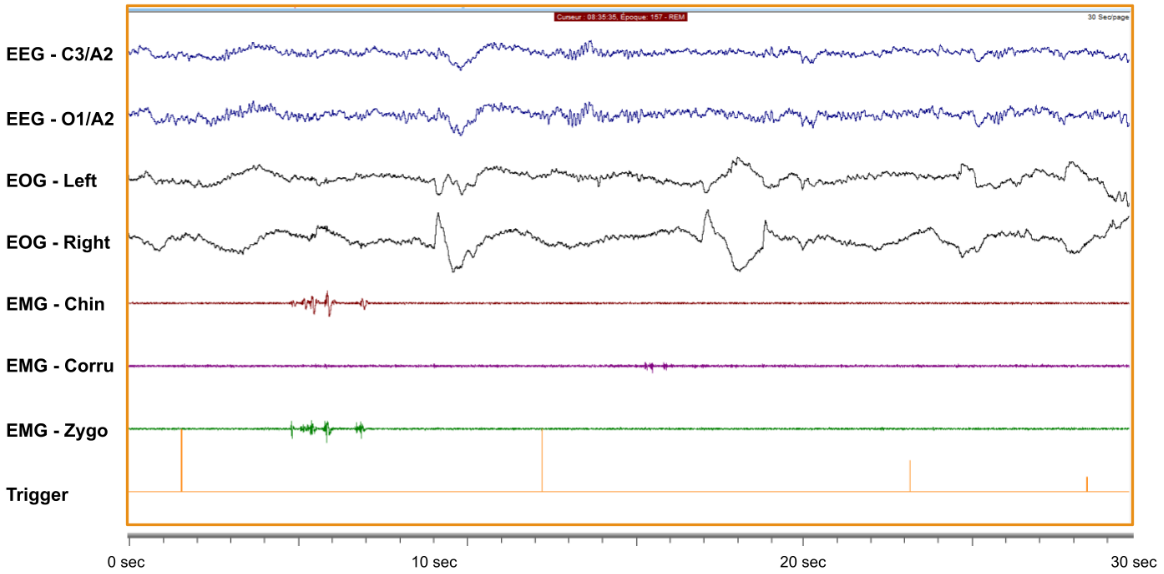

Supplementary Figure S11 Example of responses from a healthy participant during REM sleep.. All raw EEG and behavioral data are available on OSF (see Data Availability statement).

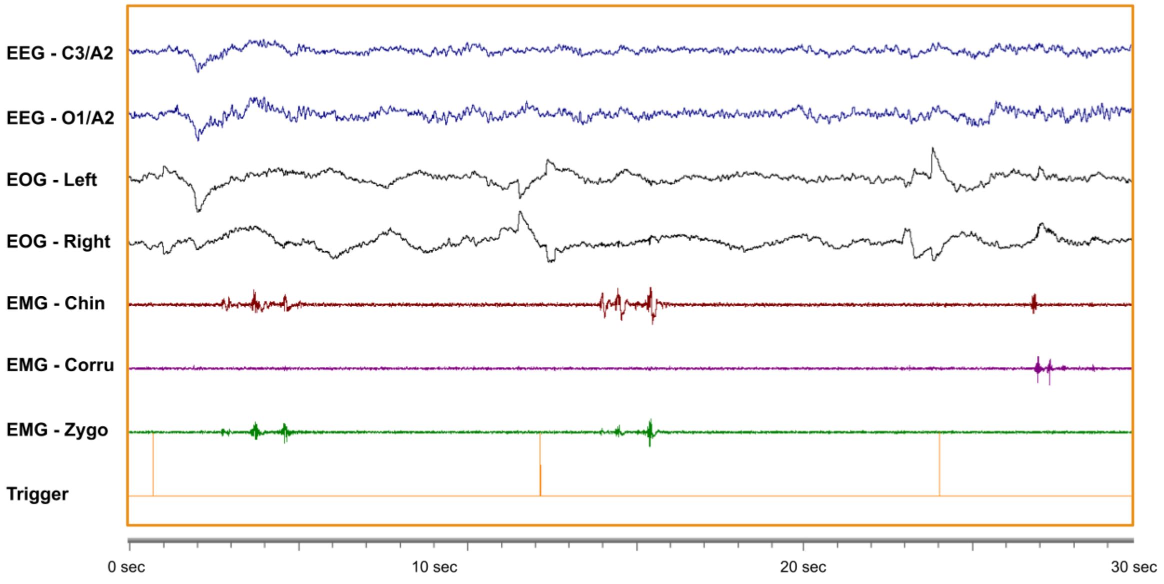

Supplementary Figure S12. Total number of correct and incorrect responses in different sleep stages.

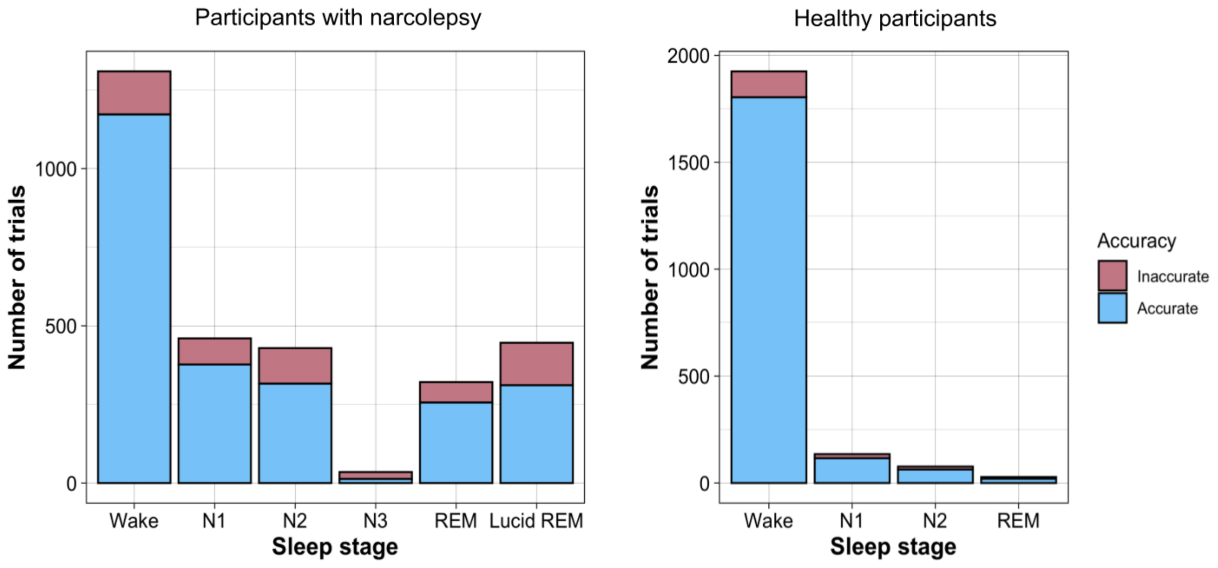

Supplementary Figure S13. Reaction times for words and pseudowords in different sleep stages in participants with narcolepsy and healthy participants.

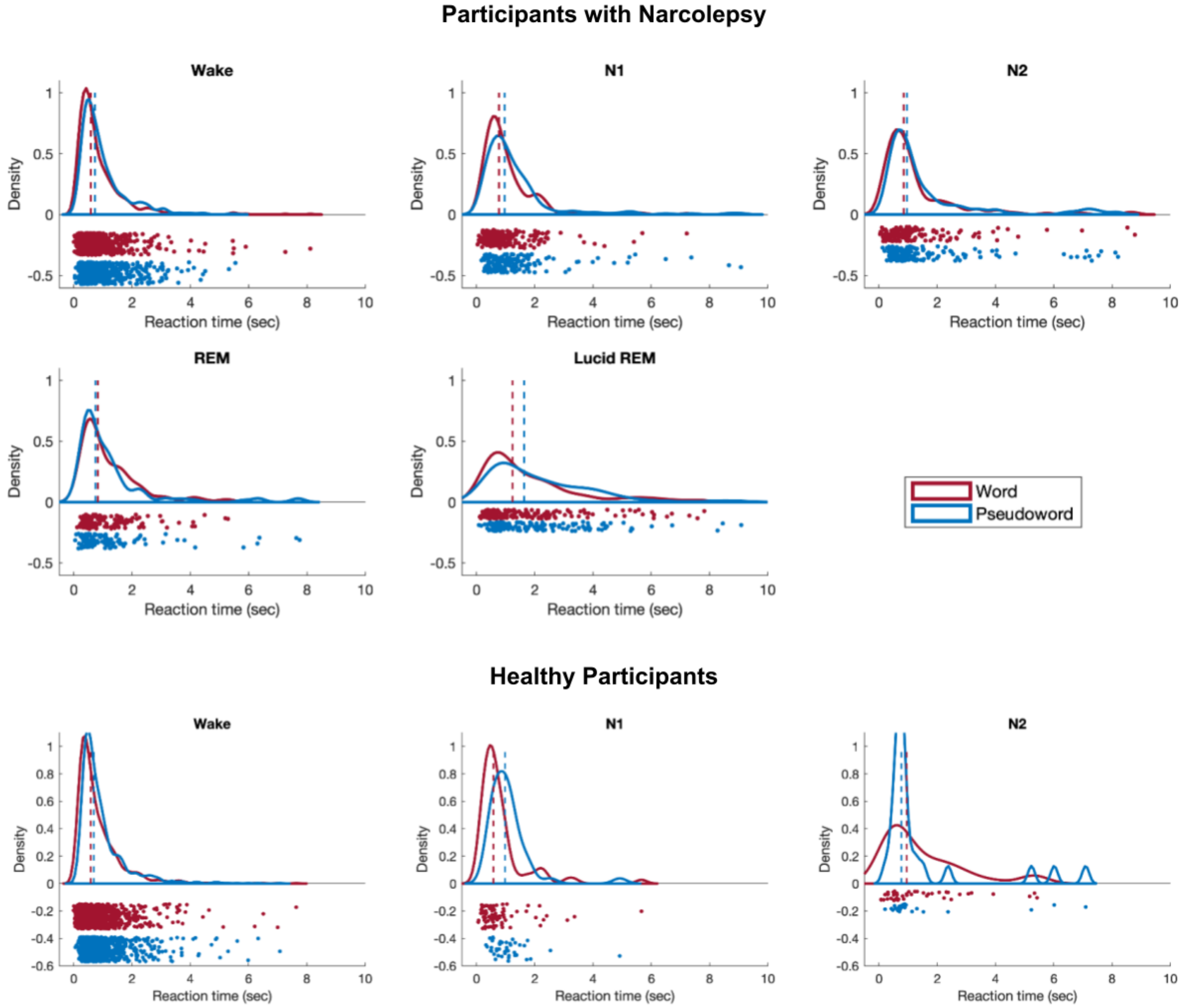

Supplementary Figure S14. Stimulus-locked ERP analysis in participants with narcolepsy. Dashed vertical lines indicate stimulus onset. Significant differences are indicated by yellow shade (FDR corrected p-value<0.05, mass univariate analysis on time dimension using mixed linear models with responsiveness as the explanatory factor and subject ID as a random effect)

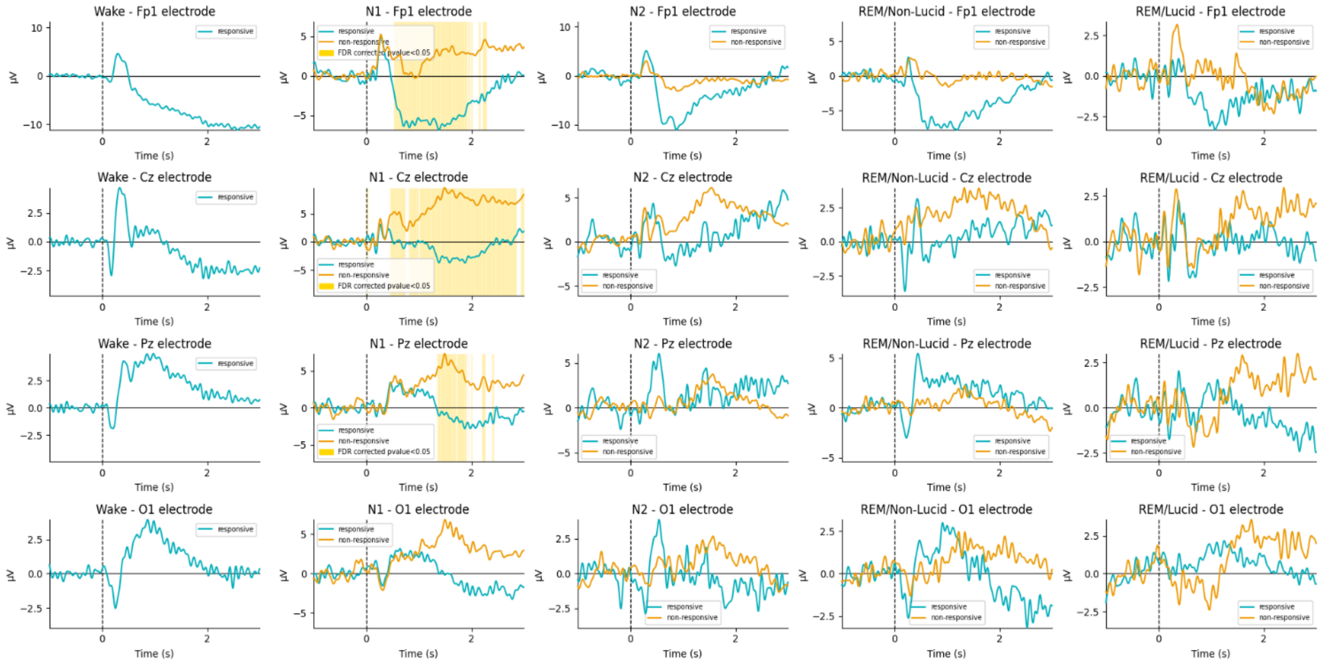

Supplementary Figure S15. F1-score of the Random Forest classifiers trained with the neurophysiological markers to classify between responsive vs non-responsive trials, in different sleep stages, for participants with narcolepsy (NP) and healthy participants (HP). Blue (true classifier): true performance of the classifier (mean F1 score and 95% confidence intervals (error bars) across folds). Orange (Chance): chance-level performance computed with 500 random permutations of the data labels (mean F1 score and 95% confidence intervals (error bars) across permutations). Both correct and incorrect trials were included in the analysis. Statistical difference between true performance and chance is assessed using 500-permutations test (NP: Wake N= 961 from 22 participants, p = 0.002; N1 N = 505 from 24 participants, p = 0.05; N2 N = 1537 from 23 participants, p = 0.002; REM sleep N = 587 from 15 participants, p = 0.002; HP: N2 N = 1339 from 20 participants, p = 0.004) Note that smallest p value obtainable via a 500-permutation procedure is 0.002.

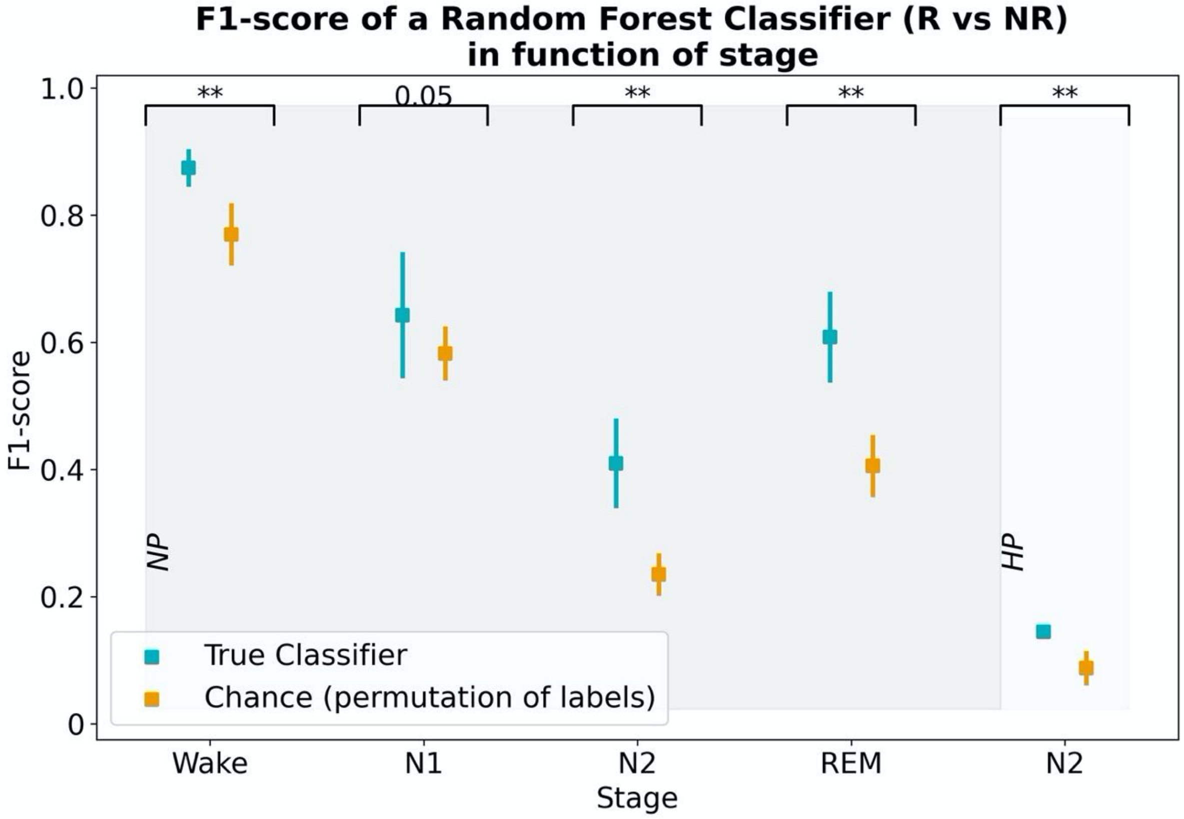

SUPPLEMENTARY TABLES

COMPARISON	Healthy Participants (HP)		Participants with Narcolepsy (NP)	
	z	FDR corrected p-value	*z*	FDR corrected p-value
**Wake - N1**	21.02	< 0.0001	13.47	< 0.0001
**Wake - N2**	31.81	< 0.0001	31.26	< 0.0001
**Wake - N3**	8.25	< 0.0001	18.20	< 0.0001
**Wake - REM**	19.27	< 0.0001	21.89	< 0.0001
**Wake - Lucid REM**	-	-	15.27	< 0.0001
**N1 - N2**	11.96	< 0.0001	19.51	< 0.0001
**N1 - N3**	5.39	< 0.0001	12.08	< 0.0001
**N1 - REM**	6.31	< 0.0001	9.57	< 0.0001
**N1 - Lucid REM**	-	-	2.15	0.03
**N2 - N3**	3.39	0.0008	4.36	< 0.0001
**N2 - REM**	-2.39	0.017	-8.54	< 0.0001
**N2 - Lucid REM**	-	-	-18.11	< 0.0001
**N3 - REM**	-3.91	0.0001	-7.55	< 0.0001
**N3 - Lucid REM**	-	-	-11.11	< 0.0001
**REM - Lucid REM**	-	-	-7.87	< 0.0001

Table S1. Details of the multiple comparisons of the response rates during ON periods in different sleep stages. Statistics are computed are computed separately for each sleep stage using generalized linear mixed model with stimulation period (ON vs. OFF) as fixed effect and subject ID as random effect. Post-hoc analyses are made via pairwise two-sided comparisons. An overall FDR correction Is applied separately to HP and NP.

Parameters	Stage	Accuracy	Chance Accuracy	P-value	Recall	Chance Recall	P-value	Precision	Chance Precision	P-value
W: 1s , Th: 5	Wake	0.79	0.49	0.002	0.93	0.57	0.002	0.69	0.42	0.002
	N1	0.81	0.70	0.002	0.83	0.25	0.002	0.32	0.09	0.002
	N2	0.75	0.73	0.002	0.60	0.26	0.002	0.08	0.03	0.002
	REM	0.76	0.71	0.002	0.77	0.27	0.002	0.14	0.05	0.002
W: 1s, Th: 7	Wake	0.82	0.50	0.002	0.90	0.52	0.002	0.73	0.42	0.002
	N1	0.86	0.74	0.002	0.79	0.19	0.002	0.40	0.09	0.002
	N2	0.81	0.79	0.002	0.56	0.19	0.002	0.10	0.03	0.002
	REM	0.82	0.78	0.002	0.68	0.19	0.002	0.17	0.05	0.002
W: 1s, Th: 10	Wake	0.83	0.50	0.002	0.87	0.48	0.002	0.76	0.42	0.002
	N1	0.89	0.77	0.002	0.75	0.16	0.002	0.46	0.09	0.002
	N2	0.87	0.84	0.002	0.50	0.13	0.002	0.13	0.05	0.002
	REM	0.86	0.81	0.002	0.68	0.15	0.002	0.22	0.05	0.002
W: 2s , Th: 5	Wake	0.820	0.50	0.002	0.90	0.51	0.002	0.74	0.42	0.002
	N1	0.86	0.74	0.002	0.8	0.20	0.002	0.4	0.09	0.002
	N2	0.83	0.79	0.002	0.58	0.18	0.002	0.11	0.03	0.002
	REM	0.82	0.78	0.002	0.68	0.19	0.002	0.17	0.04	0.002
W: 2s, Th: 7	Wake	0.83	0.50	0.002	0.86	0.48	0.002	0.77	0.42	0.002
	N1	0.89	0.77	0.002	0.75	0.16	0.002	0.46	0.09	0.002
	N2	0.87	0.85	0.002	0.50	0.13	0.002	0.14	0.04	0.002
	REM	0.88	0.83	0.002	0.68	0.14	0.002	0.24	0.05	0.002
W: 2s, Th: 10	Wake	0.83	0.51	0.002	0.81	0.43	0.002	0.79	0.42	0.002
	N1	0.91	0.79	0.002	0.71	0.13	0.002	0.54	0.10	0.002
	N2	0.91	0.88	0.002	0.45	0.09	0.002	0.18	0.04	0.002
	REM	0.91	0.86	0.002	0.57	0.10	0.002	0.28	0.05	0.002

Table S2. Different performance metrics (accuracy, recall and precision) measuring the concordance between automatic and manual scoring of responses in Wake, N1, N2 and REM sleep in healthy participants for each parameter combination [Window (W) = 1 or 2 seconds. Threshold *k* (Th) = 5, 7, 10]. Significance level of each metric against their chance-level performance computed via a 500-permutations test are also shown. Note that chance-level accuracy was not 0.5 in sleep due to the imbalance between responsive and unresponsive trials. Importantly, the smallest obtainable p-value with a 500-permutations procedure is 0.002 (1/N permutations). Note that p value are not corrected for multiple comparisons.

Parameters	Stage	Accuracy	Chance Accuracy	P-value	Recall	Chance Recall	P-value	Precision	Chance Precision	P-value
W: 1s , Th: 5	Wake	0.79	0.49	0.002	0.90	0.56	0.002	0.71	0.44	0.002
	N1	0.85	0.58	0.002	0.88	0.34	0.002	0.65	0.25	0.002
	N2	0.80	0.70	0.002	0.72	0.24	0.002	0.3	0.1	0.002
	N3	0.67	0.65	0.002	0.54	0.33	0.002	0.08	0.05	0.002
	REM	0.79	0.64	0.002	0.72	0.28	0.002	0.43	0.17	0.002
	REML	0.75	0.55	0.002	0.74	0.38	0.002	0.53	0.27	0.002
W: 1s, Th: 7	Wake	0.81	0.49	0.002	0.86	0.51	0.002	0.74	0.44	0.002
	N1	0.87	0.6	0.002	0.83	0.30	0.002	0.69	0.25	0.002
	N2	0.84	0.75	0.002	0.63	0.18	0.002	0.35	0.10	0.002
	N3	0.75	0.73	0.004	0.44	0.25	0.004	0.08	0.05	0.004
	REM	0.82	0.67	0.002	0.65	0.23	0.002	0.48	0.17	0.002
	REML	0.78	0.58	0.002	0.68	0.31	0.002	0.59	0.27	0.002
W: 1s, Th: 10	Wake	0.80	0.50	0.002	0.80	0.46	0.002	0.76	0.44	0.002
	N1	0.88	0.62	0.002	0.77	0.26	0.002	0.75	0.25	0.002
	N2	0.87	0.78	0.002	0.55	0.14	0.002	0.40	0.10	0.002
	N3	0.81	0.79	0.002	0.44	0.2	0.002	0.11	0.04	0.002
	REM	0.84	0.70	0.002	0.60	0.20	0.002	0.53	0.17	0.002
	REML	0.79	0.60	0.002	0.60	0.26	0.002	0.63	0.27	0.002
W: 2s , Th: 5	Wake	0.80	0.50	0.002	0.86	0.51	0.002	0.74	0.44	0.002
	N1	0.87	0.60	0.002	0.83	0.29	0.002	0.70	0.25	0.002
	N2	0.85	0.76	0.002	0.62	0.18	0.002	0.35	0.10	0.002
	N3	0.75	0.73	0.002	0.44	0.24	0.002	0.09	0.05	0.002
	REM	0.82	0.68	0.002	0.62	0.22	0.002	0.48	0.17	0.002
	REML	0.78	0.58	0.002	0.65	0.30	0.002	0.59	0.27	0.002
W: 2s, Th: 7	Wake	0.81	0.50	0.002	0.81	0.46	0.002	0.77	0.44	0.002
	N1	0.87	0.78	0.002	0.54	0.14	0.002	0.40	0.10	0.002
	N2	0.87	0.79	0.002	0.54	0.14	0.002	0.40	0.10	0.002
	N3	0.83	0.79	0.002	0.44	0.17	0.002	0.12	0.05	0.002
	REM	0.84	0.70	0.002	0.6	0.2	0.002	0.54	0.17	0.002
	REML	0.79	0.61	0.002	0.59	0.25	0.002	0.63	0.27	0.002
W: 2s, Th: 10	Wake	0.81	0.51	0.002	0.76	0.42	0.002	0.79	0.44	0.002
	N1	0.88	0.63	0.002	0.71	0.23	0.002	0.78	0.25	0.002
	N2	0.89	0.82	0.002	0.47	0.10	0.002	0.45	0.10	0.002
	N3	0.87	0.85	0.002	0.33	0.11	0.002	0.14	0.05	0.002
	REM	0.86	0.72	0.002	0.57	0.16	0.002	0.61	0.17	0.002
	REML	0.80	0.63	0.002	0.52	0.21	0.002	0.67	0.27	0.002

Table S3. Different performance metrics (accuracy, recall and precision) measuring the concordance between automatic and manual scoring of response in Wake, N1, N2, N3, REM and Lucid REM (REML) sleep in participants with narcolepsy for each parameter combinations [Window (W) = 1 or 2 seconds. Threshold *k* (Th) = 5, 7, 10]. Significance level of each metric against their chance-level performance computed via 500-permuations test are also shown. Note that chance-level accuracy was not 0.5 in sleep due to the imbalance between responsive and unresponsive trials. Importantly, the smallest obtainable p-value with a 500-permutations procedure is 0.002 (1/N permutations). P values are not corrected for multiple comparisons.

	Participants with Narcolepsy (NP)				Participants without Narcolepsy (HP)			
	PSD |δ|		PSD |α|		PSD |δ|		PSD |α|	
	Pre-stim	Post-stim	Pre-stim	Post-stim	Pre-stim	Post-stim	Pre-stim	Post-stim
**Wake - N1**	t =-17.84 p < .0001	t = -22.61 p < .0001	t = 24.53 p < .0001	t = 27.92 p < .0001	t = -13.68 p < .0001	t = -36.50 p < .0001	t = 21.11 p < .0001	t = 45.67 p < .0001
**Wake -N2**	t = -32.13 p < .0001	t = -34.71 p < .0001	t = 36.57 p < .0001	t = 39.84 p < .0001	t = -31.02 p < .0001	t = -76.63 p < .0001	t = -33.23 p < .0001	t = 76.13 p < .0001
**Wake - REM**	t = -23.29 p < .0001	t = -31.98 p < .0001	t = 30.05 p < .0001	t = 37.80 p < .0001	-	-	-	-

Table S4. Statistical differences of the PSD values at alpha and delta frequencies between wake and different sleep stages during pre-stimulation (-1s to 0) and post-stimulation (0 to 8s) periods. Statistics are computed using linear mixed models with sleep stage as fixed effect and subject ID as random effect at the trial level. All p values are corrected for multiple comparisons using Benjamini-Hochberg procedure following post-hoc two-sided pairwise comparisons.

	K	PE θ	SE	wSMI θ	PSD |γ|	PSD |β|	PSD |α|	PSD |θ|	PSD |δ|
**Wake - N1**	t = 9.88<br>p < .0001	t = 21.08<br>p < .0001	t = 12.37<br>p < .0001	t = 6.29<br>p < .0001	t = 9.54<br>p < .0001	t = 13.63<br>p < .0001	t = 25.91<br>p < .0001	t = -12.44<br>p < .0001	t = -17.95<br>p < .0001
**Wake -N2**	t = 27.01<br>p < .0001	t = 33.30<br>p < .0001	t = 28.90<br>p < .0001	t = 8.90<br>p < .0001	t = 17.76<br>p < .0001	t = 22.08<br>p < .0001	t = 38.66<br>p < .0001	t = -12.38<br>p < .0001	t = -32.48<br>p < .0001
**Wake - N3**	t = 44.86<br>p < .0001	t = 33.84<br>p < .0001	t = 39.14<br>p < .0001	t = 6.24<br>p < .0001	t = 19.70<br>p < .0001	t = 26.95<br>p < .0001	t = 31.16<br>p < .0001	t = -1.53<br>p = 0.14	t = -37.69<br>p < .0001
**Wake - REM**	t = 13.63<br>p < .0001	t = 24.95<br>p < .0001	t = 15.78<br>p < .0001	t = 8.08<br>p < .0001	t = 10.35<br>p < .0001	t = 14.35<br>p < .0001	t = 31.50<br>p < .0001	t = -10.51<br>p < .0001	t = -23.44<br>p < .0001
**N1 - N2**	t = 13.30<br>p < .0001	t = 6.62<br>p < .0001	t = 12.28<br>p < .0001	t = 1.06<br>p = 0.31	t = 5.41<br>p < .0001	t = 4.82<br>p < .0001	t = 6.18<br>p < .0001	t = 2.55<br>p = 0.13	t = -9.12<br>p < .0001
**N1 - N3**	t = 34.63<br>p < .0001	t = 14.63<br>p < .0001	t = 27.09<br>p < .0001	t = 0.68<br>p = 0.51	t = 10.83<br>p < .0001	t = 14.36<br>p < .0001	t = 8.07<br>p < .0001	t = 8.98<br>p < .0001	t = -20.72<br>p < .0001
**N1 - REM**	t = 3.55<br>p = .0005	t = 3.83<br>p = .0002	t = 3.29<br>p = 0.001	t = 1.71<br>p = 0.1	t = 0.90<br>p = 0.39	t = 0.88<br>p = 0.39	t = 5.48<br>p < .0001	t = -1.53<br>p = 0.14	t = -5.16<br>p < .0001
**N2 - N3**	t = 29.05<br>p < .0001	t = 11.36<br>p < .0001	t = 21.00<br>p < .0001	t = -0.15<br>p = 0.88	t = 7.93<br>p < .0001	t = 12.68<br>p < .0001	t = 3.95<br>p < .0001	t = 8.35<br>p< .0001	t = -16.32<br>p < .0001
**N2 - REM**	t = -8.91<br>p < .0001	t = -1.89<br>p = 0.06	t = -8.20<br>p < .0001	t = 1.06<br>p = 0.31	t = -4.31<br>p < .0001	t = -3.73<br>p = 0.0002	t = 0.59<br>p = 0.56	t = -0.66<br>p = 0.52	t = 2.79<br>p = 0.006
**N3 - REM**	t = -29.82<br>p < .0001	t = -10.51<br>p < .0001	t = -22.85<br>p < .0001	t = 0.90<br>p = 0.39	t = -9.53<br>p < .0001	t = -12.90<br>p < .0001	t = -2.73<br>p = 0.007	t = -7.18<br>p < .0001	t = 15.23<br>p < .0001

Table S5. Details of the multiple comparisons of neurophysiological markers in different sleep stages in NP. K for Kolmogorov Complexity; PE θ for Permutation Entropy in the theta band; SE for Sample Entropy; and wSMI θ for weighted symbolic mutual information in the theta band. Statistics are computed using linear mixed models with sleep stage as fixed effect and participant ID as random effect. All p-values are FDR corrected following post-hoc two-sided pairwise comparisons.

	K	PE θ	SE	wSMI θ	PSD |γ|	PSD |β|	PSD |α|	PSD |θ|	PSD |δ|
**Wake - N1**	t = 6.66 p < .0001	t = 17.12 p < .0001	t = 6.37 p < .0001	t = 6.59 p < .0001	t = 2.56 p = 0.013	t = 4.47 p < .0001	t = 22.52 p < .0001	t = -6.58 p < .0001	t = -13.93 p < .0001
**Wake -N2**	t = 33.86 p < .0001	t = 34.39 p < .0001	t = 34.53 p < .0001	t = 10.79 p < .0001	t = 22.36 p < .0001	t = 13.18 p < .0001	t = 25.37 p < .0001	t = -6.06 p < .0001	t = -31.23 p < .0001
**Wake - N3**	t = 38.01 p < .0001	t = 23.42 p < .0001	t = 29.93 p < .0001	t = 5.92 p < .0001	t = 13.96 p < .0001	t = 14.74 p < .0001	t = 20.26 p < .0001	t = 4.52 p < .0001	t = -26.74 p < .0001
**Wake - REM**	t = 24.34 p < .0001	t = 20.19 p < .0001	t = 26.56 p < .0001	t = 7.34 p < .0001	t = 18.79 p < .0001	t = 13.72 p < .0001	t = 21.37 p < .0001	t = -5.41 p < .0001	t = -21.65 p < .0001
**N1 - N2**	t = 18.67 p < .0001	t = 8.46 p < .0001	t = 19.50 p < .0001	t = 1.29 p = 0.22	t = 14.20 p < .0001	t = 5.36 p < .0001	t = 3.77 p = 0.0002	t = 2.13 p = 0.04	t = -9.28 p < .0001
**N1 - N3**	t = 30.44 p < .0001	t = 10.39 p < .0001	t = 23.25 p < .0001	t = 1.14 p = 0.28	t = 11.10 p < .0001	t = 10.59 p < .0001	t = 4.05 p < .0001	t = 8.35 p < .0001	t = -15.46 p < .0001
**N1 - REM**	t = 15.32 p = .0009	t = 3.08 p = 0.003	t = 17.48 p < .0001	t = 0.69 p = 0.53	t = 13.98 p < .0001	t = 8.03 p < .0001	t = -0.36 p = 0.73	t = 0.84 p = 0.43	t = -6.93 p < .0001
**N2 - N3**	t = 21.20 p < .0001	t = 5.72 p < .0001	t = 12.41 p < .0001	t = 0.37 p = 0.74	t = 2.39 p = 0.02	t = 8.20 p < .0001	t = 1.89 p = 0.07	t = 7.99 p < .0001	t = -10.91 p < .0001
**N2 - REM**	t = 0.03 p = 0.98	t = -5.00 p < .0001	t = 1.96 p = 0.06	t = -0.48 p = 0.66	t = 3.03 p = 0.003	t = 4.72 p < .0001	t = -4.46 p < .0001	t = -1.17 p = 0.27	t = 0.87 p = 0.42
**N3 - REM**	t = -19.41 p < .0001	t = -8.36 p < .0001	t = -10.15 p < .0001	t = -0.64 p = 0.55	t = -0.29 p = 0.78	t = -4.57 p < .0001	t = -4.51 p < .0001	t = -8.05 p < .0001	t = 10.54 p < .0001

Table S6. Details of the multiple comparisons of neurophysiological markers in different sleep stages in HP. K for Kolmogorov Complexity; PE θ for Permutation Entropy in the theta band; SE for Sample Entropy; and wSMI θ for weighted symbolic mutual information in the theta band. Statistics are computed using linear mixed models with sleep stage as fixed effect and participant ID as random effect. All p-values are FDR corrected following post-hoc two-sided pairwise comparisons.

Participants with Narcolepsy (NP)									
	**K**	**PE θ**	**SE**	**wSMI θ**	**PSD |γ|**	**PSD |β|**	**PSD |α|**	**PSD |θ|**	**PSD |δ|**
**Wake**	t = 2.63<br>p = 0.024	t = 0.14<br>p = 0.96	t = 3.15<br>p = 0.007	t = -0.22<br>p = 0.93	t = 2.55<br>p = 0.025	t = 0.84<br>p = 0.53	t = 0.65<br>p = 0.66	t = -0.07<br>p = 0.99	t = -1.18<br>p = 0.35
**N1**	t = 4.52<br>p < .0001	t = 1.48<br>p = 0.21	t = 3.87<br>p = 0.0006	t = -0.01<br>p = 0.99	t = 2.60<br>p = 0.025	t = 2.57<br>p = 0.025	t = 2.30<br>p = 0.043	t = -0.63<br>p = 0.66	t = -4<br>p = 0.0005
**N2**	t = 4.94<br>p < .0001	t = 1.85<br>p = 0.11	t = 5.03<br>p < .0001	t = -0.51<br>p = 0.71	t = 5.32<br>p < .0001	t = 2.28<br>p = 0.043	t = 0.56<br>p = 0.69	t = -0.86<br>p = 0.53	t = -1.47<br>p = 0.21
**REM**	t = 3.91<br>p = 0.0006	t = 2.63<br>p = 0.02	t = 3.5<br>p = 0.002	t = 1.62<br>p = 0.18	t = 2.34<br>p = 0.041	t = 2.94<br>p = 0.012	t = 1.87<br>p = 0.11	t = 0.05<br>p = 0.99	t = -2.93<br>p = 0.012
**Healthy Participants (HP)**									
	**K**	**PE θ**	**SE**	**wSMI θ**	**PSD |γ|**	**PSD |β|**	**PSD |α|**	**PSD |θ|**	**PSD |δ|**
**Wake**	t = 2.42<br>p = 0.048	t = 3.78<br>p = 0.005	t = 1.26<br>p = 0.40	t = -0.46<br>p = 0.76	t = 0.21<br>p = 0.87	t = 2.32<br>p = 0.056	t = 2.67<br>p = 0.035	t =-2.44<br>p = 0.048	t = -1.97<br>p = 0.12
**N1**	t = 3.29<br>p = 0.007	t = 0.90<br>p = 0.55	t = 2.70<br>p = 0.035	t = 0.99<br>p = 0.51	t = 1.71<br>p = 0.2	t = 1.36<br>p = 0.36	t = -0.36<br>p = 0.78	t = -0.08<br>p = 0.94	t = -0.71<br>p = 0.61
**N2**	t = 2.46<br>p = 0.048	t = 0.65<br>p = 0.63	t = 3.29<br>p = 0.007	t = -1.22<br>p = 0.40	t = 3.27<br>p = 0.007	t = 1.06<br>p = 0.49	t = -0.41<br>p = 0.77	t = 0.76<br>p = 0.60	t = -0.86<br>p = 0.55

Table S7 Statistical differences of the neurophysiological markers between responsive and non-responsive trials in different sleep stages, in non-lucid naps, for HP and NP. K for Kolmogorov Complexity; PE θ for Permutation Entropy in the theta band; SE for Sample Entropy; and wSMI θ for weighted symbolic mutual information in the theta band. Statistics are computed separately for each sleep stage using linear mixed models with responsiveness as fixed effect and participant ID as random effect. All p-values are FDR corrected for multiple comparisons separately for each participant group (combining all sleep stages) following post-hoc two-sided pairwise comparisons.

.

		Participants with narcolepsy (NP)		Healthy participants (HP)	
**Marker**	**Sleep Stage**	**Responsive trials**	**Unresponsive trials**	**Responsive trials**	**Unresponsive trials**
**K**	**Wake**	0.6803754	0.6759375	0.6759382	0.6781734
	**N1**	0.6715431	0.6657330	0.6719169	0.6690935
	**N2**	0.6608676	0.6540851	0.6524095	0.6439975
	**N3**	0.6184315	0.6201406	-	-
	**REM**	0.6641869	0.6586848	0.6477605	0.6458158
**PE θ**	**Wake**	0.9438212	0.9607482	0.9553089	0.9487543
	**N1**	0.9089780	0.9061032	0.9178698	0.9242548
	**N2**	0.9068446	0.9024781	0.9057451	0.9034864
	**N3**	0.8785161	0.8788696	-	-
	**REM**	0.9110372	0.9046189	0.9275586	0.9045421
**SE**	**Wake**	0.7667154	0.7137313	0.7226942	0.7604313
	**N1**	0.6969705	0.6614606	0.6936492	0.7003899
	**N2**	0.6314248	0.5938238	0.5807554	0.5415817
	**N3**	0.4410244	0.4680347	-	-
	**REM**	0.6326181	0.6070131	0.5471580	0.5503706
**wSMI θ**	**Wake**	0.05851454	0.06273602	0.06149339	0.06386605
	**N1**	0.05114335	0.05127260	0.05559300	0.05590678
	**N2**	0.05047093	0.05062645	0.05073267	0.05295049
	**N3**	0.05035443	0.04943042	-	-
	**REM**	0.05267189	0.05029265	0.05458694	0.05269153
**PSD |δ|**	**Wake**	0.3194139	0.2605850	0.3072278	0.3533609
	**N1**	0.4503646	0.5030020	0.4671423	0.4538805
	**N2**	0.5119116	0.5370847	0.5442573	0.5576962
	**N3**	0.7045447	0.6870387	-	-
	**REM**	0.5025181	0.5284724	0.5086240	0.5643875
**PSD |θ|**	**Wake**	0.2068933	0.1418656	0.2007460	0.1939149
	**N1**	0.2656362	0.2414224	0.2475708	0.2244246
	**N2**	0.2575809	0.2429896	0.2323852	0.2305370
	**N3**	0.2000923	0.1947102	-	-
	**REM**	0.2399718	0.2332216	0.2438726	0.2417497
**PSD |α|**	**Wake**	0.25245023	0.39240107	0.31084653	0.2446519
	**N1**	0.11708552	0.10398341	0.12343722	0.1485939
	**N2**	0.10530406	0.09841525	0.09927345	0.1027312
	**N3**	0.05095843	0.06146279	-	-
	**REM**	0.11324187	0.10276048	0.15921852	0.1004059
**PSD |β|**	**Wake**	0.1699562	0.17224221	0.14886467	0.16731716
	**N1**	0.1332042	0.12198210	0.13141251	0.13987564
	**N2**	0.1032444	0.10267684	0.11045073	0.09906883
	**N3**	0.0383426	0.04763254	-	-
	**REM**	0.1207973	0.11569344	0.07990251	0.08175934
**PSD |γ|**	**Wake**	0.05128644	0.032906174	0.032315047	0.040755087
	**N1**	0.03370946	0.029610126	0.030437162	0.033225455
	**N2**	0.02195909	0.018833625	0.013633360	0.009966815
	**N3**	0.00606192	0.009155819	-	-
	**REM**	0.02347083	0.019852047	0.008382299	0.011697608

Table S8. Average values of neurophysiological markers in different sleep stages in NP and HP. K for Kolmogorov Complexity; PE θ for Permutation Entropy in the theta band; SE for Sample Entropy; and wSMI θ for weighted symbolic mutual information in the theta band. Note that these are non z-scored trial averages. For the statistical comparisons (Figure 5A and Table S5), trial values were z-scored and estimated marginal means were computed. Figure 5A shows the differences between the estimated means of responsive and non-responsive trials.

Participants with Narcolepsy (NP)						
	True positives (TP)	False positives (FP)	True negatives (TN)	False negatives (FN)	Balanced accuracy	F1 score
Wake	671 (80.1%)	33 (25.2%)	98 (74.8%)	159 (19.2%)	0.78 (78%)	0.87
N1	181 (59%)	72 (36.4%)	126 (63.6%)	126 (41%)	0.61 (61%)	0.64
N2	187 (67%)	448 (35.6%)	810 (64.4%)	92 (33%)	0.66 (66%)	0.41
REM	151 (65.9%)	101 (28.2%)	257 (71.8%)	78 (34.1%)	0.67 (67%)	0.61
Healthy Participants (HP)						
	True positives (TP)	False positives (FP)	True negatives (TN)	False negatives (FN)	Balanced accuracy|	F1 score
N2	29 (39.7%)	318 (25.1%)	948 (74.9%)	44 (60.3%)	0.58 (58%)	0.14

Table S9. Confusion matrix and performance scores of the random forest classifier (responsive vs non-responsive trials) in different sleep stages, for HP and NP.

Lucid REM Sleep	K	PE θ	SE	wSMI θ	PSD |γ|	PSD |β|	PSD |α|	PSD |θ|	PSD |δ|
Responsive vs. Non-responsive trials	*t* = 1.26<br>*p* = 0.62<br>BF = 0.17	*t* = 0.05<br>*p* = 0.96<br>BF = 0.08	*t* = 1.18<br>*p* = 0.53<br>BF = 0.18	*t* = -0.58<br>*p* = 0.75<br>BF = 0.10	*t* = 0.55<br>*p* = 0.75<br>BF = 0.10	*t* = -1.31<br>*p* = 0.53<br>BF = 0.21	*t* = -0.70<br>*p* = 0.75<br>BF = 0.11	*t* = 1.20<br>*p* = 0.53<br>BF = 0.17	*t* = -0.06<br>*p* = 0.96<br>BF = 0.08

Table S10. Statistical differences of the neurophysiological markers between responsive and non-responsive trials in lucid REM sleep (participants with narcolepsy). K for Kolmogorov Complexity; PE θ for Permutation Entropy in the theta band; SE for Sample Entropy; and wSMI θ for weighted symbolic mutual information in the theta band. Frequentist statistics are computed using linear mixed models with responsiveness as fixed effect and participant ID as random effect. All p-values are FDR corrected following post-hoc two-sided pairwise comparisons. BF: Bayes Factor of the comparison between the full model (response + subject identity) and a null model (subject identity only).

Lucid REM sleep vs. REM sleep	K	PE θ	SE	wSMI θ	PSD |γ|	PSD |β|	PSD |α|	PSD |θ|	PSD |δ|
All trials	*t* = 2.08<br>*p* = 0.067	*t* = 2.33<br>*p* = 0.045	*t* = 2.99<br>*p* = 0.024	*t* = -1.13<br>*p* = 0.33	*t* = 2.73<br>*p* = 0.024	*t* = 1.90<br>*p* = 0.087	*t* = 0.96<br>*p* = 0.38	*t* = 0.72<br>*p* = 0.47	*t* = -2.66<br>*p* = 0.024
Responsive trials	*t* = 1.49<br>*p* = 0.31<br>BF = 0.17	*t* = -0.07<br>*p* = 0.94<br>BF = 0.14	*t* = 1.97<br>*p* = 0.31<br>BF = 0.28	*t* = -1.62<br>*p* = 0.31<br>BF = 0.17	*t* = 1.55<br>*p* = 0.31<br>BF = 0.21	*t* = -0.36<br>*p* = 0.81<br>BF = 0.19	*t* = -0.74<br>*p* = 0.69<br>BF = 0.14	*t* = 1.37<br>*p* = 0.31<br>BF = 0.21	*t* = -0.54<br>*p* = 0.76<br>BF = 0.20

Table S11. Statistical differences of the neurophysiological markers between lucid and non-lucid trials (REM sleep), for all trials and for responsive trials only. K for Kolmogorov Complexity; PE θ for Permutation Entropy in the theta band; SE for Sample Entropy; and wSMI θ for weighted symbolic mutual information in the theta band. Frequentist statistics are computed using linear mixed models with lucidity (lucid or not) as fixed effect and participant ID as random effect. All p-values are FDR corrected following post-hoc two-sided pairwise comparisons. BF: Bayes Factor of the comparison between the full model (response + subject identity) and a null model (subject identity only).

Group	Nap	Wake		N1		N2		N3		REM		Sleep Latency		Micro-arousals		TST		Micro-arousal Index		WASO		Efficiency	
		%	std	%	std	%	std	%	std	%	std	min	std	number	std	min	std	number/H	std	min	std	%	std
Participants with Narcolepsy	Nap 1	10	21.21	16.33	17.85	24.55	23.7	2.63	9.44	46.5	31.57	2.86	4.65	11.85	8.37	15.7	5.51	52.05	50.56	1.43	3.32	90	21.21
	Nap 2	4.86	14.65	25.06	23.46	40.73	24.7	7.02	15.17	21.88	30.34	3.33	4.63	9.8	7.76	16.01	5.16	36.85	29.16	0.66	1.67	95.14	14.65
	Nap 3	5.05	7.06	24.74	23.3	41.03	24.03	13.33	22.48	15.85	28.61	3.48	4.01	9.19	7.08	15.77	4.23	39.73	35.44	0.75	0.99	94.95	7.06
	Nap 4	8.3	13.93	18.47	15.75	42.16	28.44	11.93	21.01	19.15	30.76	2.31	2.44	10.7	6.74	16.28	3.59	42.43	29.5	1.41	2.49	91.7	13.93
	Nap 5	7.58	13.31	19.49	15.85	26.04	22.41	7.91	21.7	38.97	34.12	4.22	4.19	9.79	7.09	14.73	4.77	38.67	32.56	1.05	1.87	92.42	13.31
	Total	7.158	14.032	20.818	19.242	34.902	24.656	8.564	17.96	28.47	31.08	3.24	3.984	10.266	7.408	15.698	4.652	41.946	35.444	1.06	2.068	92.842	14.032
Healthy Participants	Morning N = 14	18.82	18.08	20.69	7.39	36.63	14.04	10.07	13.7	13.79	12.13	16.52	13.02	35.36	15.23	69.5	21.98	33.65	16.3	13.98	10.89	81.18	18.08
	Afternoon N = 8	42.37	18.43	26.64	18.07	29.18	25.44	1.81	4.71	0	0	18.02	25.83	36.75	15.12	47.04	22.32	57.61	39.16	34.94	19.51	57.63	18.43
	Total	27.38	21.21	22.85	12.3	33.92	18.74	7.07	11.84	8.78	11.71	17.07	18.11	35.86	14.84	61.33	24.24	42.36	28.54	21.6	17.52	72.62	21.21

Table S12. Detailed information on the sleep characteristics of the participants with narcolepsy and healthy participants.
