# Supplementary material for: Behavioral and brain responses to verbal stimuli reveal transient periods of cognitive integration of the external world during sleep
Source: Nat Neurosci. 2023 Oct 12;26(11):1981–93. doi: 10.1038/s41593-023-01449-7 (PMC10620087; doi:10.1038/s41593-023-01449-7)
Supplement: Supplementary file 2 — Reporting Summary [file 41593_2023_1449_MOESM2_ESM.pdf]

nature portfolio

Corresponding author(s): Delphine Oudiette

Last updated by author(s): 2023, August 1

Reporting Summary

Nature Portfolio wishes to improve the reproducibility of the work that we publish. This form provides structure for consistency and transparency in reporting. For further information on Nature Portfolio policies, see our Editorial Policies and the Editorial Policy Checklist.

Statistics

For all statistical analyses, confirm that the following items are present in the figure legend, table legend, main text, or Methods section.

n/a	Confirmed	
☐	☒	The exact sample size (*n*) for each experimental group/condition, given as a discrete number and unit of measurement
☐	☒	A statement on whether measurements were taken from distinct samples or whether the same sample was measured repeatedly
☐	☒	The statistical test(s) used AND whether they are one- or two-sided<br>*Only common tests should be described solely by name; describe more complex techniques in the Methods section.*
☐	☒	A description of all covariates tested
☐	☒	A description of any assumptions or corrections, such as tests of normality and adjustment for multiple comparisons
☐	☒	A full description of the statistical parameters including central tendency (e.g. means) or other basic estimates (e.g. regression coefficient) AND variation (e.g. standard deviation) or associated estimates of uncertainty (e.g. confidence intervals)
☐	☒	For null hypothesis testing, the test statistic (e.g. *F*, *t*, *r*) with confidence intervals, effect sizes, degrees of freedom and *P* value noted<br>*Give P values as exact values whenever suitable.*
☐	☒	For Bayesian analysis, information on the choice of priors and Markov chain Monte Carlo settings
☐	☒	For hierarchical and complex designs, identification of the appropriate level for tests and full reporting of outcomes
☐	☒	Estimates of effect sizes (e.g. Cohen's *d*, Pearson's *r*), indicating how they were calculated

Our web collection on statistics for biologists contains articles on many of the points above.

Software and code

Policy information about availability of computer code

Data collection	Stimuli were presented through speakers using the Psychtoolbox extension for MATLAB (The MathWorks). EEG data were amplified through a Grael 4K PSG:EEG amplifier (Medical Data Technology, Compumedics Ltd, Australia).
Data analysis	Sleep stages were scored offline using Profusion software (COMPUMEDICS, Medical Data Technology); spindles and slow waves were computed using the YASA (v0.6.3) algorithm. For the pre-processing of EEG data, we used the MNE (v 1.4.2) package and the Autoreject algorithm (v 0.4.2). Most statistical analyses were conducted in R using lme4, emmeans, BayesFactor, car, and DHARMa packages. For the machine learning analysis, statistics were conducted in Python using the numpy (v 1.22.1), scipy (1.9.1), pingouin (0.5.3), and scikit-learn packages (1.3).<br>Examples of custom analysis scripts can be found in OSF (https://osf.io/gbtjd/?view_only=254e0addb97a4b108e2fe35cce076799).

For manuscripts utilizing custom algorithms or software that are central to the research but not yet described in published literature, software must be made available to editors and reviewers. We strongly encourage code deposition in a community repository (e.g. GitHub). See the Nature Portfolio guidelines for submitting code & software for further information.

Data

Policy information about availability of data

All manuscripts must include a data availability statement. This statement should provide the following information, where applicable:

- Accession codes, unique identifiers, or web links for publicly available datasets
- A description of any restrictions on data availability
- For clinical datasets or third party data, please ensure that the statement adheres to our policy

All data that support the findings of the study can be found in OSF (https://osf.io/gbtjd/?view_only=254e0addb97a4b108e2fe35cce076799).

Research involving human participants, their data, or biological material

Policy information about studies with human participants or human data. See also policy information about sex, gender (identity/presentation), and sexual orientation and race, ethnicity and racism.

Reporting on sex and gender	During the recruitment process, we seek to ensure an equal representation of male and female participants (participants' sex was self-reported). We did not conduct a sex-based analysis of our results as we did not have specific hypotheses about putative differences and such analyses would reduce statistical power.
Reporting on race, ethnicity, or other socially relevant groupings	During the recruitment process, we did not ask volunteers to report their race, ethnicity, or other socially relevant information.
Population characteristics	See below
Recruitment	Healthy participants were recruited via an internet survey. Participants who met the selection criteria (e.g., adults, no sleep disorders, native french speakers, no hearing disability; ability to nap easily) were randomly selected. Note that the database from which we selected participants include a large proportion of young adults (<35 y.o) interested in cognitive experiments and might thus not represent the entire diversity of the general population.<br>Patients with narcolepsy were recruited from the database of the the National Reference Center for Narcolepsy in Pitié-Salpêtrière University Hospital. The clinicians pre-selected patients based on their ability to lucid dream. All these patients were contacted and the available ones participated in the study.<br>Participants with and without narcolepsy were paid €200 and €70 respectively, as compensation for their participation in the study (participants with narcolepsy also took part in a second experiment, not described here).<br>All participants gave their written consent to participate to the study.
Ethics oversight	The protocol had been approved by the local ethics committee (CPP Ile-de-France 8).

Note that full information on the approval of the study protocol must also be provided in the manuscript.

Field-specific reporting

Please select the one below that is the best fit for your research. If you are not sure, read the appropriate sections before making your selection.

☐ Life sciences ☒ Behavioural & social sciences ☐ Ecological, evolutionary & environmental sciences

For a reference copy of the document with all sections, see nature.com/documents/nr-reporting-summary-flat.pdf

Behavioural & social sciences study design

All studies must disclose on these points even when the disclosure is negative.

Study description	In this study, participants were invited to nap while hearing words or pseudo-words verbally delivered via a speaker. Whenever they heard a stimulus, be it while awake or while sleeping, they were instructed to frown or smile twice depending on the stimulus type (e.g. frowning for word or smiling for pseudo-word). Participants' sleep/wake state was constantly monitored via polysomnography, and behavioral responses to stimuli were assessed via corrugator (frowning response) and zygomatic (similing response) EMG.
Research sample	Participants with narcolepsy: Thirty participants with narcolepsy were recruited for this study (14 women, mean age: 35 ± 11 years) from the patients followed in the National Reference Center for Narcolepsy in Pitié-Salpêtrière University Hospital.<br>Healthy participants: Twenty-two healthy participants (all non-lucid dreamers) were recruited for this study (10 women, mean age: 24 ± 4 years).<br>We chose to include participants with narcolepsy because: 1) we first aimed to examine behavioral responsiveness in an admitted conscious sleep state, -lucid dreaming-, because we reckoned that it would be the sleep stage during which behavioral responses are the most likely to happen given our previous results (Konkoly et al., Current Biology 2021) and 2) lucid dreaming is more frequent and easier to capture in a laboratory setting in this population (Oudiette et al., Sci Reports 2018). Healthy participants were recuited as

well to test the generalisibility of behavioral responsiveness beyond lucid REM sleep in narcolepsy. We randomly select healthy participants who met the inclusion criteria from an internet survey. This procedure should increase the representativity of our sample, but is restricted to young individuals living in France.

Sampling strategy	Healthy participants were recruited via an internet survey. Participants who met the selection criteria (e.g. no sleep disorders, native french speakers, no hearing disability; ability to nap easily) were randomly selected. Patients with narcolepsy were recruited from the database of the the National Reference Center for Narcolepsy in Pitié-Salpêtrière University Hospital. The clinicians pre-selected patients based on their ability to lucid dream. All these patients were contacted and the available ones participated in the study. No statistical methods were used to pre-determine sample sizes but our sample sizes were similar or higher to those reported in previous studies investigating sensory processing during sleep (Strauss et al., PNAS 2015; Andrillon et al., J Neuro 2016). Furthermore, our design included repeated measures within participants, thus increasing statistical power. We used mixed model with subjects as random effect to take into account inter-subject variability.
Data collection	Behavioral data were collected via the Psychtoolbox extension for MATLAB (The MathWorks) on a computer. EEG data were collected through a Grael 4K PSG:EEG amplifier (Medical Data Technology, Compumedics Ltd, Australia). Dream reports were collected through a microphone and recorded on a computer. The researcher(s) in charge of the data collection were not blind to the group and were aware of the study hypotheses.
Timing	The data were collected from 9th of May 2019 to 9th of March 2021
Data exclusions	Data from 4 participants were discarded from the analyses because of technical issues affecting the recordings
Non-participation	No participants dropped out
Randomization	Single condition, no randomization

Reporting for specific materials, systems and methods

We require information from authors about some types of materials, experimental systems and methods used in many studies. Here, indicate whether each material, system or method listed is relevant to your study. If you are not sure if a list item applies to your research, read the appropriate section before selecting a response.

Materials & experimental systems

n/a	Involved in the study
☒	☐ Antibodies
☒	☐ Eukaryotic cell lines
☒	☐ Palaeontology and archaeology
☒	☐ Animals and other organisms
☒	☐ Clinical data
☒	☐ Dual use research of concern
☒	☐ Plants

Methods

n/a	Involved in the study
☒	☐ ChIP-seq
☒	☐ Flow cytometry
☒	☐ MRI-based neuroimaging
